# Supplementary material for: Prevalence and comorbidity of anxiety disorder in school-attending children and adolescents aged 6–16 years in China
Source: BMJ Paediatr Open. 2024 Mar 27;8(1):e001967. doi: 10.1136/bmjpo-2023-001967 (PMC10982779; doi:10.1136/bmjpo-2023-001967)
Supplement: Supplementary data [file bmjpo-2023-001967supp001.pdf]

Supplementary File 1. The prevalence rate of anxiety disorder at different age.

Anxiety disorder \ Age	6	7	8	9	10	11	12	13	14	15	16
Panic disorder	0.3	0.1	0.3	0	0.1	0.2	0.5	0.5	0.2	0.1	0
Agoraphobia without panic	0.1	0	0.1	0	0.1	0.2	0.3	0.3	0	0	0
Separation anxiety disorder	0.1	0.4	2.4	1.7	1.3	0	0.1	0.1	0.2	0.1	0.1
Social phobia	0	0.9	0.3	0.7	0.3	1.1	1.2	1.5	1.1	0.7	0.5
Specific phobia	0.4	0.3	0.7	0.4	0	0.1	0.1	0	0	0	0.1
Generalized Anxiety Disorder	0.4	0.2	1.1	0.9	2.7	1.5	0.6	0.4	1.1	2.5	2.2
Total	1.4	2.0	5.0	3.8	4.4	3.1	2.8	2.8	2.6	3.4	3.0
