# Supplementary material for: Prevalence and comorbidity of anxiety disorder in school-attending children and adolescents aged 6–16 years in China
Source: BMJ Paediatr Open. 2024 Mar 27;8(1):e001967. doi: 10.1136/bmjpo-2023-001967 (PMC10982779; doi:10.1136/bmjpo-2023-001967)
Supplement: Author's manuscript [file bmjpo-2023-001967.draft_revisions.pdf]

BMJ Paediatrics Open

BMJ Paediatrics Open is committed to open peer review. As part of this commitment we make the peer review history of every article we publish publicly available.

When an article is published we post the peer reviewers' comments and the authors' responses online. We also post the versions of the paper that were used during peer review. These are the versions that the peer review comments apply to.

The versions of the paper that follow are the versions that were submitted during the peer review process. They are not the versions of record or the final published versions. They should not be cited or distributed as the published version of this manuscript.

BMJ Paediatrics Open is an open access journal and the full, final, typeset and author-corrected version of record of the manuscript is available on our site with no access controls, subscription charges or pay-per-view fees (http://bmjpaedsopen.bmj.com).

If you have any questions on BMJ Paediatrics Open's open peer review process please email info.bmjpo@bmj.com

BMJ Paediatrics Open

Prevalence and comorbidity of anxiety disorder in school-attending children and adolescents aged 6-16 in China

Journal:	*BMJ Paediatrics Open*
Manuscript ID	bmjpo-2023-001967
Article Type:	Original research
Date Submitted by the Author:	30-May-2023
Complete List of Authors:	Wang, Fang; National Center for Children Healthy, Department of Psychiatry<br>Yang, Hanxue; Beijing Language and Culture University<br>Li, Fenghua; Chinese Academy of Sciences<br>Zheng, Yi; Capital Medical University, Department of pediatrics<br>Xu, Hui; National Center for Children's Health<br>Wang, Rui; National Center for Children’s Health<br>Li, Ying; National Center for Children Healthy, Department of Psychiatry<br>Cui, Cui; National Center for Children's Health, Department of Psychiatry
Keywords:	Adolescent Health, Child Psychiatry, Epidemiology

SCHOLARONE™
Manuscripts

I, the Submitting Author has the right to grant and does grant on behalf of all authors of the Work (as defined in the below author licence), an exclusive licence and/or a non-exclusive licence for contributions from authors who are: i) UK Crown employees; ii) where BMJ has agreed a CC-BY licence shall apply, and/or iii) in accordance with the terms applicable for US Federal Government officers or employees acting as part of their official duties; on a worldwide, perpetual, irrevocable, royalty-free basis to BMJ Publishing Group Ltd ("BMJ") its licensees and where the relevant Journal is co-owned by BMJ to the co-owners of the Journal, to publish the Work in this journal and any other BMJ products and to exploit all rights, as set out in our licence.

The Submitting Author accepts and understands that any supply made under these terms is made by BMJ to the Submitting Author unless you are acting as an employee on behalf of your employer or a postgraduate student of an affiliated institution which is paying any applicable article publishing charge ("APC") for Open Access articles. Where the Submitting Author wishes to make the Work available on an Open Access basis (and intends to pay the relevant APC), the terms of reuse of such Open Access shall be governed by a Creative Commons licence – details of these licences and which Creative Commons licence will apply to this Work are set out in our licence referred to above.

Other than as permitted in any relevant BMJ Author's Self Archiving Policies, I confirm this Work has not been accepted for publication elsewhere, is not being considered for publication elsewhere and does not duplicate material already published. I confirm all authors consent to publication of this Work and authorise the granting of this licence.

Confidential: For Review Only

Prevalence and comorbidity of anxiety disorder in school-attending children and adolescents aged 6-16 in China

Fang Wang[1], Hanxue Yang[2], Fenghua Li[3], Yi Zheng[4], Hui Xu[5], Rui Wang [6], Ying Li[1*] Yonghua Cui[1*]

1, Department of Psychiatry, Beijing Children's Hospital, Capital Medical University, National Center for Children Healthy, Beijing, China.

2, School of Psychology, Beijing Language and Culture University, Beijing, China

3, Key Lab of Mental Health, Institute of Psychology, Chinese Academy of Sciences, Beijing, China

4, Department of pediatrics, Beijing Anding Hospital, Capital Medical University, Beijing, China

5, Big Data Center, Beijing Children's Hospital, Capital Medical University, National Center for Children's Health, Beijing, China

6, Laboratory of Nutrition and Development, Key Laboratory of Major Diseases in Children, Ministry of Education, Beijing Pediatric Research Institute, Beijing Children's Hospital, Capital Medical University, National Center for Children's Health, Beijing, China

Yonghua Cui, Beijing Children's Hospital, Capital Medical University, National Center for Children Healthy, 56 Nanlishi Road, Beijing 100101, China; Email: cuiyonghua@bch.com.cn; Tel/Fax number: +86(0)10-59616161

Ying Li, Beijing Children's Hospital, Capital Medical University, National Center for Children Healthy, 56 Nanlishi Road, Beijing 100101, China; Email: liying@bch.com.cn; Tel/Fax number: +86(0)10-59616161

Confidential: For Review Only

Abstract:

Background: Anxiety disorder is common in the pediatric population. As an internal symptom, it may not be recognized in time by caregivers, which prevents children and adolescents with anxiety disorders from accessing medical services. In China, there are no data on the national prevalence of pediatric anxiety disorders and its subtypes.

Methods: A nationwide epidemiological survey of mental disorders in school students aged 6-16 years old was conducted. Participants were randomly recruited from five provinces in China. The Child Behavior Checklist was used to screen students at high risk for mental disorder. The final diagnosis was made based on the Diagnostic and Statistical Manual-IV. The point prevalence and comorbidity rate of anxiety disorder were calculated, and the difference between age, sex, and socioeconomic status groups was also compared.

Results: Generalized anxiety disorder (GAD) was the most common anxiety disorder in school-attending children and adolescents, with a prevalence rate of 1.3%, (95% CI: 1.2%-1.3%). Separation anxiety and specific phobia were more common in children than in adolescents. Girls had a higher prevalence of panic disorder (0.3% vs. 0.2%, $\chi^2 = 14.6$, $p < 0.001$) and agoraphobia (0.9% vs. 0.8%, $\chi^2 = 4.3$, $p = 0.03$) than boys. We found no significant difference between developed and less developed areas. Girls were more likely to have panic disorder and GAD than boys, with ratios of 2.13:1 and 1.01:1, respectively. The co-occurrence of anxiety disorder and attention-deficit and disruptive behavior disorder was very common, ranging from 40% to 85%.

Conclusions: This national epidemiological survey showed that anxiety disorder was common among school students in China. The prevalence rate was influenced by gender and age but not by socioeconomic factors. Comorbidity was very common for anxiety disorder, highlighting the need for comprehensive assessment in clinical practice.

Keywords: anxiety disorder, prevalence, children and adolescents, comorbidity

Confidential: For Review Only

1. Introduction

Anxiety disorder are characterized by excessive anxiety, worry, and related behaviors. Anxiety disorders, including panic disorder, generalized anxiety disorder (GAD), specific phobia, separation anxiety disorder, specific disorder and agoraphobia, and social phobia, are common in the pediatric population[1–5]. Comorbidity with other mental disorders, such as major depressive disorder and substance abuse, is very common in anxiety disorder[6,7]. Anxiety disorder and its comorbidities not only cause great distress to children, adolescents and their families but also have a negative impact on their educational achievement and social function in adulthood[8–10]. Furthermore, anxiety disorders increase the risk of other mental disorders[11]. Children with anxiety disorder were more likely to have depression disorder, suicidal behavior, and other psychiatric disorders in adulthood [12].As anxiety is an internal symptom, caregivers and teachers may not recognize the onset of anxiety in children and adolescents, which would prevent children and adolescents with anxiety disorders from accessing medical services[13,14]. It would therefore be of great importance to know the prevalence and characteristics of anxiety disorder in school settings in order to adapt our policies and provide psychological services to children and adolescents.

There have been many surveys exploring the prevalence of anxiety disorder in the pediatric population. However, the results vary widely between countries, ranging from 2% to 31%[6,15–19]. The prevalence of anxiety disorder was 6.3% in children aged 7-10 years and 4% in adolescents aged 11-17 years in a German national survey including 17641 participants[15]. A national survey in the United States including 10123 adolescents reported that the lifetime prevalence was as high as 31.9%[18]. Another recent study reported a lifetime prevalence of 15.6% among Austrian adolescents[17]. The difference might come from several aspects. First, the age range of the population included was different. Second, the setting of the study was also different. Most epidemiological studies were conducted in school settings[17,20,21], but some studies selected participants from community samples[22] or mental health institutions[17]. And third, the screening tools and diagnostic criteria were also different[21,23]. Some considered functional impairment when making the diagnosis, while others did

Confidential: For Review Only

not[20,24].Comorbidity with other mental disorders was also very common in anxiety disorder, and the results were also inconsistent. The coexistence of different subtypes of anxiety disorder was 17-60%[16,25]. The comorbid rate of anxiety disorder and depression disorder was reported to be 10%-40%[26,27]. The co-occurrence rates of anxiety disorder and attention-deficit hyperactivity disorder (ADHD) was similar in different studies. The comorbid rate was about 17-25%[16,28]. The prevalence of conduct disorder was also high in patients with anxiety disorder (18-38%)[5,16].

In China, several epidemiological studies have investigated the prevalence of anxiety disorders in the pediatric population. A previous survey conducted in a single province, including 20752 6- to 16-year-old students, reported that the 6-month prevalence rate of anxiety disorder was 3.94%[23]. Chen et al. investigated 10118 children aged 7-12 years from primary and middle schools in Taiwan and reported that the lifetime prevalence of anxiety disorder was 15.2%[21]. The prevalence of any anxiety disorder among adolescents in Hong Kong was estimated to be 30.2%[20]. However, all of these studies were conducted locally, which limits the representativeness of the results. At present, there are no nationwide epidemiological data on anxiety disorders in the pediatric population.

Our group recently reported the first national prevalence study of mental disorders in school-aged children and adolescents in China[29]. This was a two-phase epidemiological survey of schoolchildren aged 6– 16 years, and the diagnostic criteria were based on the DSM-IV. This study was a further analysis of the data collected in the two-phase national epidemiological survey. The aim was to investigate the prevalence of anxiety disorder subtypes, their comorbid conditions, and the distribution profile between different ages, gender, and locations.

2. Method

2.1 Included Participants

This is a two-stage survey. Detailed information, including the sampling design, sample size calculation, and socio-demographic characteristics of the sampling areas included , has been described in previous studies(see Supplementary Materials). In

Confidential: For Review Only

summary, the survey was carried out in Beijing, Liaoning, Jiangsu, Sichuan, and Hunan Provinces. These five regions were different in terms of geographical partition, economic development, and rural/urban factors and could be considered as good representatives of developed and developing areas in China. Multistage cluster sampling was applied in the above mentioned five areas. The details of the multistage cluster sampling were described in our previous study[29].

2.2 CBCL screening

In the first stage, the Child Behavior Checklist (CBCL) was used for screening. The Chinese version of the CBCL has been shown to have good reliability and validity in clinical use[30]. For all the enrolled students, their parents or other caregivers were asked to complete the CBCL, and the total problem score was calculated. According to the previous study of Liu et al., we chose a cut-off score of 35, which was the 90th percentile of the CBCL Total Problems score in their study[31]. Students with CBCL Total Problems scores higher than 35 (including 35) were identified as high-risk and underwent the second-stage interview.

2.3 MINI-KID and DSM-IV interview

This stage was performed approximately two weeks after the completion of the first stage. Considering the false-negative rate of CBCL screening, we also randomly selected 5% of the participants who showed CBCL-negative results and included them in the stage 2 assessment. They and the high-risk individuals underwent detailed interviews using the Mini-International Neuropsychiatric Interview for Children and Adolescents (MINI-KID). The MINI-KID is a widely used structured interview tool for the diagnosis of psychiatric disorders in children and adolescents[32]. In our study, the student and his or her parent or caregiver were interviewed at the same time. They also underwent the DSM-IV interview. Psychiatrists who performed the DSM-IV interview referred to the results of the MINI-KID rating, but the final diagnosis was made based on the DSM-IV criteria. The study procedure is illustrated in Figure 1.

Figure 1. Flowchart

Confidential: For Review Only

2.4 Ethical approval

Informed consent was obtained from all the participants and/or their parents. The protocol was interviewed and approved by the Ethics Committee of the Beijing Anding Hospital, Capital Medical University.

2.5 Statistical analysis

R software 3.4.3 was used for statistical analysis. The point prevalence of different subtypes of anxiety disorder and their comorbidity rates were calculated. The prevalence was adjusted based on the weights of their provincial region, prefectural division, county/district, school, and class. Taylor series linearization was used to calculate the 95% confidence intervals (CI) of the prevalence rate. The locations included in this study were classified into developed areas (Beijing) and developing areas (Liaoning, Jiangsu, Sichuan, and Hunan provinces) based on economic development conditions. The prevalence of anxiety disorder was also compared between age, gender, and socio-economic condition groups using Rao-Scott adjusted chi-square tests. All statistical tests were two-sided tests, and $P < 0.05$ was considered significant.

3. Result

3.1. Characteristics of the included population

In total, 1764 classes from 81 primary schools and 88 middle schools (with 73992 students) were randomly selected. Finally, 71929 individuals completed the CBCL screening in stage 1, and 17524 individuals (14653 high-risk individuals and 2871 randomly selected non-high-risk individuals) underwent stage 2 assessment. The mean age of the included population was 11.56 ± 5.12 years, and 66.2% lived in urban areas. The sex ratio of boys/girls was 0.99:1. Eighteen percent of the total sample were diagnosed with at least one mental disorder. Among those who were not at high risk, 5.5% were diagnosed with at least one mental disorder.

3.2 Point prevalence of anxiety disorder

The point prevalence of different subtypes of anxiety disorder and their 95% confidence intervals are shown in Table 1. The most prevalent anxiety disorder in children and adolescents was GAD, with an adjusted point prevalence of 1.3% (95% CI: 1.2%-1.3%). Followed by social phobia and separation anxiety disorder, the adjusted point prevalence was 0.8% (95% CI: 0.8% –1.0%) and 0.6% (95% CI: 0.5% –1.0%), respectively. The adjusted point prevalence of panic disorder was 0.2% (95% CI: 0.2%-0.2%). A total of 0.2% (95% CI: 0.1%-0.2%) were diagnosed with specific phobia, and 0.1% (95% CI: 0.1%-0.1%) were diagnosed with agoraphobia.

3.3 The effect of age, gender, and socio-economic conditions on the prevalence of anxiety disorder

As listed in Table 2, the distribution of anxiety disorder subtypes differed between different age groups. Adolescents had a higher rates of panic disorder, GAD, agoraphobia, and social phobia than children. Separation anxiety disorder and specific phobia were more common in children than in adolescents. The prevalence rate of separation anxiety was 0.90% (95% CI: 0.81%-0.99%) in children and 0.15% (95% CI: 0.11%-0.20%) in adolescents. ($\chi 2 = 282.5, p < 0.001$). Specific phobia had a significantly higher rate in the child group. (0.23% vs 0.05%, $\chi 2 = 55.5$, $p < 0.001$) As shown in Figure 2, age had a significant effect on the prevalence of different subtypes of anxiety disorder. The prevalence of anxiety disorder was relatively low before the age of 8 years old. For different subtypes of anxiety disorder, the peak prevalence age for separation anxiety disorder was between 7 and 10 years old, and the prevalence was relatively low after 11 years old. The prevalence of social phobia rises sharply after the age of 10 years old.

The prevalence of the panic disorder, GAD, and agoraphobia were significantly different between gender groups. The prevalence of panic disorder in girls was one time higher than in boys. In the group of boys, the prevalence rate was 0.15 % (95% CI: 0.11%-0.19%), while in the group of girls it was 0.32% (95% CI: 0.26%-0.38%, $\chi^2 = 14.6$, $p < 0.001$). Girls also had a slightly higher rates of GAD and agoraphobia than boys. The prevalence rate of agoraphobia was 0.08% (95% CI: 0.05%-0.10%) in

Confidential: For Review Only

the boy group and 0.14% (95% CI: 0.11%-0.19%) in the girl group. ($\chi^2 = 4.3$, p = 0.03). For GAD, the prevalence rate was 1.27% (95% CI: 1.16%-1.39%) in the girl group and 1.25% (95% CI: 1.13%-1.36%) in the boy group. ($\chi2 = 4.71$, p = 0.02). There were no significant differences in the prevalence of specific phobia, social phobia, and separation anxiety between boys and girls (for details, please see Table 3).

Based on the socioeconomic conditions, the five areas included in the study were divided into developed and underdeveloped areas. There was no significant difference in the prevalence of any anxiety disorder between these two groups. The results are listed in Table 4.

Insert table 2-4 and figure 2

3.4 Comorbid conditions of anxiety disorder

A total of 70.3% (95% CI: 68.4%- 72.2%) of the children were diagnosed with anxiety disorder comorbid with at least one other mental disorder. A total of 42.2% (95% CI: 40.2%- 44.3%) had comorbidity with one other mental disorder, 27.5% (95% CI: 25.7%- 29.4%) were comorbid with two other mental disorders, and 0.6% (95% CI: 0.3%- 1.0%) were comorbid with three other mental disorders. The co-occurrence of anxiety disorder and ADHD, conduct disorder (CD) and oppositional defiant disorder (ODD) was very common. The most common comorbidity of GAD, specific phobia, and separation anxiety disorder was ODD, with comorbid rates of 26.9%, 74.7%, and 49%, respectively. The most common comorbidity for panic disorder was CD (24%). The ADHD-inattention subtype ranks as the most prevalent comorbidity for agoraphobia. Tic disorder was also common in anxiety disorder, and the comorbid rate of provisional tic disorder was 5.2% for GAD, 7.2% for panic disorder, and 10.8% for specific phobia. Tourette's syndrome and chronic vocal tic disorder were also common in agoraphobia. The concurrent rates were 10.1% and 6.3%, respectively. In our survey, there was no comorbidities of anxiety disorder and eating disorder, adjustment disorder, psychotic disorders, or pervasive developmental disorder (for details, please refer to figure 3).

Confidential: For Review Only

Insert Figure 3

4. Discussion

Our group had completed the first national survey carried out in China to investigate the prevalence of mental disorders in school students aged 6–16 years old[29]. This study was a further analysis of the previous findings. Our results showed that GAD was the most common anxiety disorder in school children and adolescents, and with a prevalence rate of 1.3%, followed by social phobia (0.9%), separation anxiety disorder (0.6%), panic disorder (0.2%), specific phobia (0.2%), and agoraphobia (0.1%). The prevalence of separation anxiety disorder and specific phobia was higher in children than in adolescents. Compared to children, adolescents had higher rates of panic disorder, GAD and agoraphobia. Girls were more likely to be affected by panic disorder and GAD than boys, with ratios of 2.13:1 and 1.01:1, respectively. In terms of comorbidity, the co-occurrence of anxiety disorder and attention-deficit and disruptive behavior disorders was very common, with rates ranging from 40% to 85%. Tic disorders and depressive disorders were also commonly observed in the pediatric anxiety population.

Previous studies have shown that the prevalence of anxiety disorders in the pediatric population varies from 2% to 31% in different countries[15–18,21,23]. The basic information of these studies and the results are listed in Supplementary Table 1. As we mentioned in the introduction, the difference may be due to the sample source, screening and diagnostic tools, and other factors. The prevalence of any anxiety disorder in our study was 4.7%. Our result was compatible with two other surveys carried out in Hunan and Sichuan provinces in China[16,23]. The prevalence rate was lower than that in many other studies[18,20,21]. The main reason might be related to the diagnostic criteria. In our survey, the DSM-IV criteria were used for the final diagnosis, which required functional impairment when making the diagnosis. A previous study showed that the prevalence of psychiatric disorders decreased sharply when the impairment criteria were applied[20,24]. In addition, stigma might be associated with low prevalence, as people in Asian countries are not used to reporting

Confidential: For Review Only

emotional problems[33,34]. Children and adolescents with self-stigma might not report their anxious experience[35]. As anxiety is an internal symptom, it might be ignored by caregivers.

Among the subtypes of anxiety disorder, GAD was the most prevalent anxiety disorder in our study (1.3%), which was consistent with two studies carried out in Hunan and Sichuan provinces, China. By investigating 17,071 primary and middle school students in Central Hunan Province, Shen et al reported that the 12-month prevalence of GAD was 1.77%, followed by 0.74% for a specific phobia, 0.07% for social phobia, and 0.02% for social anxiety disorder[16]. Roberts et al reported that agoraphobia was the most common anxiety disorder in their sample[24]. Several other studies reported that specific anxiety disorders had a much higher prevalence rate than other anxiety disorders[18,21]. However, the prevalence of panic disorder was relatively low (0.2%) in our study and other studies[18,23]. Notably, panic attacks are very common in the pediatric population. In the survey by Essau et al, 18% of adolescents reported experiencing panic attacks. However, only 0.5% met the diagnosis of panic disorder[6].

Both age and gender had effects on the prevalence of any anxiety disorder. Our results showed that girls were more likely to be affected by GAD and panic disorder. Gender differences have been reported in many previous studies[15,16,23]. Wagner et al reported that the prevalence rate of anxiety disorder was one time higher in girls than in boys (19.53 and 9.52%, respectively)[17]. However, some other studies found no gender difference[3,4]. The distribution of anxiety disorders was also different in different age groups. In general, adolescents had a higher rate of anxiety disorder, except for separation anxiety disorder. This was consistent with previous findings that anxiety disorder was less prevalent in younger children[18,36]. In our study, the peak age for separation anxiety disorder was between 7 and 10 years old. The finding was similar to that of Mohammadi et al[3]. This might be related to the fact that at this time, children separated from their parents and started attending school. Most previous studies have shown that young children have a higher prevalence rate of separation anxiety disorder[3,4]. In contrast, the prevalence of social anxiety disorder increases

with age[36]. Our results found no difference between developed areas and less developed areas. The effect of socioeconomic factors on the prevalence of anxiety disorder is not clear. A cross-nation survey indicated that socioeconomic conditions might be associated with the differences in the prevalence of anxiety subtypes, and high-income countries had higher prevalence rates of social anxiety disorder than low- and lower-middle income countries[2]. Many other studies failed to find differences between different socioeconomic status groups[17,23].

In our study, 79.8% of children with anxiety disorder had at least one comorbidity. Previous studies indicated that the comorbid rate of anxiety disorder was high[37]. The comorbidity of other subtypes of anxiety disorder, depressive disorder, ADHD and disruptive, impulse control and conduct disorders was common[4,17,36]. Our results showed that the most prevalent comorbidities of anxiety disorder were disruptive, impulse control and conduct disorders. The most common comorbidity for GAD, specific phobia and separation anxiety disorder was ODD. CD ranked as the most common comorbidity for panic disorder. The high rate of comorbidity might be due to the following reasons. First, the symptoms of anxiety disorder and ODD and CD were highly associated[38–40]. Anger or irritability symptoms in the ODD lead to anxiety[41]. In addition, the functional impairment caused by ODD increased the risk of anxiety[42]. The concurrence of anxiety disorder and disruptive, impulse control and conduct disorders would cause more severe functional impairment for the individual [43]. Comorbidity with ADHD was also very common in our study. The comorbidity of anxiety disorder and ADHD could be explained by the attentional control theory[44]. Individuals in an anxious state have increased attention to threat-related stimuli and impaired attentional inhibition and shifting skills.[45]. Executive attention was impaired in children with anxiety disorders[46]. A neuroimaging study also confirmed deficits in prefrontal attentional control mechanisms in anxiety patients[47].

The study had several limitations. First, the participants were enrolled in schools. Children who did not attend school were not included, and some of them might may not attend school because of anxiety. This would undermine the representativeness of our sample. Second, the MINI-KID and the DSM-IV criteria were used for the

Confidential: For Review Only

diagnosis. However, the validity of the MINI-KID for the diagnosis of affective disorders, such as dysthymia, was not as good[32]. This may make the comorbidity results less comprehensive. Thirdly, it should be noted that all diagnoses were made retrospectively, and recall bias could be a confounding factor for the results.

This study was a two-stage, national epidemiological survey using a stratified cluster sampling method. The participants represented school children and adolescents from different socio-economic backgrounds. The results demonstrated that anxiety disorder was common among school students. Girls were more likely to be affected by anxiety disorder than boys. The distribution of anxiety changed with age. Caregivers and teachers should pay more attention to the risk emotions of children. Our results also revealed that ADHD, ODD and CD often co-occurred with anxiety disorder. This implies that for children existing external symptoms such as aggressive symptoms and hyperactivity, the assessment of internal symptoms should not be neglected. A comprehensive assessment should be carried out in case the anxiety disorder is masked by external symptoms.

Funding statement: This work was supported by the National Natural Science Foundation of China (NSFC) under Grant No. 82001445, No. 82171538 and the Natural Science Foundation of Beijing Municipality under Grant No. 7212035, Beijing Hospitals Authority Youth Programme Grant No. QML20211203.

Competing interests: The authors declare no competing interests.

Roles of authors : Yi Zheng and Yonghua Cui was responsible for the design and carry-out of the survey. Fang Wang contributed to the data analysis and writing of the manuscript. Hui Xu and Ying Li collaborated in the writing of the manuscript. Rui Wang, Fenghua Li and Ying Li participated in collecting data collection and digitalizing the data.

Confidential: For Review Only

Reference :

1 Kessler RC, Berglund P, Demler O, *et al.* Lifetime prevalence and age-of-onset distributions of DSM-IV disorders in the National Comorbidity Survey Replication. *Arch Gen Psychiatry* 2005;**62**:593–602. doi:10.1001/archpsyc.62.6.593

2 Stein DJ, Lim CCW, Roest AM, *et al.* The cross-national epidemiology of social anxiety disorder: Data from the World Mental Health Survey Initiative. *BMC Med* 2017;**15**:143. doi:10.1186/s12916-017-0889-2

3 Mohammadi MR, Ahmadi N, Yazdi FR, *et al.* Prevalence, comorbidity and predictors of anxiety disorders among children and adolescents. *Asian J Psychiatry* 2020;**53**:102059. doi:10.1016/j.ajp.2020.102059

4 Spence SH, Zubrick SR, Lawrence D. A profile of social, separation and generalized anxiety disorders in an Australian nationally representative sample of children and adolescents: Prevalence, comorbidity and correlates. *Aust N Z J Psychiatry* 2018;**52**:446–60. doi:10.1177/0004867417741981

5 Ghandour RM, Sherman LJ, Vladutiu CJ, *et al.* Prevalence and Treatment of Depression, Anxiety, and Conduct Problems in US Children. *J Pediatr* 2019;**206**:256-267.e3. doi:10.1016/j.jpeds.2018.09.021

6 Essau CA, Conradt J, Petermann F. Frequency, comorbidity, and psychosocial impairment of anxiety disorders in German adolescents. *J Anxiety Disord* 2000;**14**:263–79. doi:10.1016/s0887-6185(99)00039-0

7 Jacobson NC, Newman MG. Anxiety and depression as bidirectional risk factors for one another: A meta-analysis of longitudinal studies. Psychol Bull. 2017 Nov;143(11):1155-1200. doi: 10.1037/bul0000111

8 Towe-Goodman NR, Franz L, Copeland W, *et al.* Perceived family impact of preschool anxiety disorders. *J Am Acad Child Adolesc Psychiatry* 2014;**53**:437–46. doi:10.1016/j.jaac.2013.12.017

9 Van Ameringen M, Mancini C, Farvolden P. The impact of anxiety disorders on educational achievement. *J Anxiety Disord* 2003;**17**:561–71. doi:10.1016/s0887-6185(02)00228-1

10 Hossain B, Bent S, Hendren R. The association between anxiety and academic performance in children with reading disorder: A longitudinal cohort study. *Dyslexia Chichester Engl* 2021;**27**:342–54. doi:10.1002/dys.1680

11 Shek DTL, Chai W, Tan L. The relationship between anxiety and depression under the pandemic: The role of life meaning. *Front Psychol* 2022;**13**:1059330. doi:10.3389/fpsyg.2022.1059330

12 Woodward LJ, Fergusson DM. Life course outcomes of young people with anxiety

Confidential: For Review Only

disorders in adolescence. *J Am Acad Child Adolesc Psychiatry* 2001;**40**:1086–93. doi:10.1097/00004583-200109000-00018

13 Koet LBM, Bennenbroek JJM, Bruggeman AYS, *et al.* GPs' and practice nurses' views on their management of paediatric anxiety problems: an interview study. *BMC Prim Care* 2022;**23**:235. doi:10.1186/s12875-022-01802-y

14 Factors associated with detection and receipt of treatment for youth with depression and anxiety disorders - PubMed. https://pubmed.ncbi.nlm.nih.gov/20129479/ (accessed 10 Jan 2023).

15 Ravens-Sieberer U, Wille N, Erhart M, *et al.* Prevalence of mental health problems among children and adolescents in Germany: results of the BELLA study within the National Health Interview and Examination Survey. *Eur Child Adolesc Psychiatry* 2008;**17 Suppl 1**:22–33. doi:10.1007/s00787-008-1003-2

16 Shen Y-M, Chan BSM, Liu J-B, *et al.* The prevalence of psychiatric disorders among students aged 6~ 16 years old in central Hunan, China. *BMC Psychiatry* 2018;**18**:243. doi:10.1186/s12888-018-1823-7

17 Wagner G, Zeiler M, Waldherr K, *et al.* Mental health problems in Austrian adolescents: a nationwide, two-stage epidemiological study applying DSM-5 criteria. *Eur Child Adolesc Psychiatry* 2017;**26**:1483. doi:10.1007/s00787-017-0999-6

18 Merikangas KR, He J-P, Burstein M, *et al.* Lifetime prevalence of mental disorders in U.S. adolescents: results from the National Comorbidity Survey Replication--Adolescent Supplement (NCS-A). *J Am Acad Child Adolesc Psychiatry* 2010;**49**:980–9. doi:10.1016/j.jaac.2010.05.017

19 Costello EJ, Mustillo S, Erkanli A, *et al.* Prevalence and Development of Psychiatric Disorders in Childhood and Adolescence. *Arch Gen Psychiatry* 2003;**60**:837. doi:10.1001/archpsyc.60.8.837

20 Leung PWL, Hung S, Ho T, *et al.* Prevalence of DSM-IV disorders in Chinese adolescents and the effects of an impairment criterion: a pilot community study in Hong Kong. *Eur Child Adolesc Psychiatry* 2008;**17**:452–61. doi:10.1007/s00787-008-0687-7

21 Chen Y-L, Chen WJ, Lin K-C, *et al.* Prevalence of DSM-5 mental disorders in a nationally representative sample of children in Taiwan: methodology and main findings. *Epidemiol Psychiatr Sci* 2019;**29**:e15. doi:10.1017/S2045796018000793

22 Vicente B, de la Barra F, Saldivia S, *et al.* Prevalence of child and adolescent psychiatric disorders in Santiago, Chile: a community epidemiological study. *Soc Psychiatry Psychiatr Epidemiol* 2012;**47**:1099–109. doi:10.1007/s00127-011-0415-3

Confidential: For Review Only

23 Qu Y, Jiang H, Zhang N, *et al.* Prevalence of Mental Disorders in 6–16-Year-Old Students in Sichuan Province, China. *Int J Environ Res Public Health* 2015;**12**:5090–107. doi:10.3390/ijerph120505090

24 Roberts RE, Roberts CR, Xing Y. Rates of DSM-IV Psychiatric Disorders Among Adolescents in a Large Metropolitan Area. *J Psychiatr Res* 2007;**41**:959–67. doi:10.1016/j.jpsychires.2006.09.006

25 Cognitive behavioral therapy, sertraline, or a combination in childhood anxiety - PubMed. https://pubmed.ncbi.nlm.nih.gov/18974308/ (accessed 17 Feb 2023).

26 Depression and Anxiety in Hong Kong during COVID-19 - PubMed. https://pubmed.ncbi.nlm.nih.gov/32466251/ (accessed 17 Feb 2023).

27 Depression and generalized anxiety disorder: cumulative and sequential comorbidity in a birth cohort followed prospectively to age 32 years - PubMed. https://pubmed.ncbi.nlm.nih.gov/17548747/ (accessed 17 Feb 2023).

28 Comorbidity between ADHD and anxiety disorders across the lifespan - PubMed. https://pubmed.ncbi.nlm.nih.gov/31232613/ (accessed 17 Feb 2023).

29 Li F, Cui Y, Li Y, *et al.* Prevalence of mental disorders in school children and adolescents in China: diagnostic data from detailed clinical assessments of 17,524 individuals. 2021;:13.

30 Test-retest reliability and criterion validity of the Chinese version of CBCL, TRF, and YSR - PubMed. https://pubmed.ncbi.nlm.nih.gov/16930392/ (accessed 13 Jan 2023).

31 Liu X, Kurita H, Guo C, *et al.* Prevalence and risk factors of behavioral and emotional problems among Chinese children aged 6 through 11 years. *J Am Acad Child Adolesc Psychiatry* 1999;**38**:708–15. doi:10.1097/00004583-199906000-00018

32 Reliability and validity of the Mini International Neuropsychiatric Interview for Children and Adolescents (MINI-KID) - PubMed. https://pubmed.ncbi.nlm.nih.gov/20331933/ (accessed 10 Jan 2023).

33 Lynch H, McDonagh C, Hennessy E. Social Anxiety and Depression Stigma Among Adolescents. *J Affect Disord* 2021;**281**:744–50. doi:10.1016/j.jad.2020.11.073

34 Dubreucq J, Plasse J, Franck N. Self-stigma in Serious Mental Illness: A Systematic Review of Frequency, Correlates, and Consequences. *Schizophr Bull* 2021;**47**:1261–87. doi:10.1093/schbul/sbaa181

35 Calear AL, Batterham PJ, Torok M, *et al.* Help-seeking attitudes and intentions for

Confidential: For Review Only

generalised anxiety disorder in adolescents: the role of anxiety literacy and stigma. *Eur Child Adolesc Psychiatry* 2021;**30**:243–51. doi:10.1007/s00787-020-01512-9

36 Cummings CM, Caporino NE, Kendall PC. Comorbidity of anxiety and depression in children and adolescents: 20 years after. *Psychol Bull* 2014;**140**:816–45. doi:10.1037/a0034733

37 Rapee RM, Lyneham HJ, Hudson JL, *et al.* Effect of comorbidity on treatment of anxious children and adolescents: results from a large, combined sample. *J Am Acad Child Adolesc Psychiatry* 2013;**52**:47–56. doi:10.1016/j.jaac.2012.10.002

38 Ezpeleta L, Granero R, de la Osa N, *et al.* Dimensions of oppositional defiant disorder in 3-year-old preschoolers. *J Child Psychol Psychiatry* 2012;**53**:1128–38. doi:10.1111/j.1469-7610.2012.02545.x

39 Humphreys KL, Aguirre VP, Lee SS. Association of anxiety and ODD/CD in children with and without ADHD. *J Clin Child Adolesc Psychol Off J Soc Clin Child Adolesc Psychol Am Psychol Assoc Div 53* 2012;**41**:370–7. doi:10.1080/15374416.2012.656557

40 Drabick DAG, Gadow KD, Loney J. Co-occurring ODD and GAD symptom groups: source-specific syndromes and cross-informant comorbidity. *J Clin Child Adolesc Psychol Off J Soc Clin Child Adolesc Psychol Am Psychol Assoc Div 53* 2008;**37**:314–26. doi:10.1080/15374410801955862

41 Drabick DAG, Gadow KD. Deconstructing oppositional defiant disorder: clinic-based evidence for an anger/irritability phenotype. *J Am Acad Child Adolesc Psychiatry* 2012;**51**:384–93. doi:10.1016/j.jaac.2012.01.010

42 Evans SC, Burke JD, Roberts MC, *et al.* Irritability in child and adolescent psychopathology: An integrative review for ICD-11. *Clin Psychol Rev* 2017;**53**:29–45. doi:10.1016/j.cpr.2017.01.004

43 Martín V, Granero R, Ezpeleta L. Comorbidity of oppositional defiant disorder and anxiety disorders in preschoolers. *Psicothema* 2014;**26**:27–32. doi:10.7334/psicothema2013.75

44 Eysenck MW, Derakshan N, Santos R, *et al.* Anxiety and cognitive performance: attentional control theory. *Emot Wash DC* 2007;**7**:336–53. doi:10.1037/1528-3542.7.2.336

45 Ironside M, Browning M, Ansari TL, *et al.* Effect of Prefrontal Cortex Stimulation on Regulation of Amygdala Response to Threat in Individuals With Trait Anxiety. *JAMA Psychiatry* 2019;**76**:71–8. doi:10.1001/jamapsychiatry.2018.2172

46 Mogg K, Salum GA, Bradley BP, *et al.* Attention network functioning in children with anxiety disorders, attention-deficit/hyperactivity disorder and non-clinical

Confidential: For Review Only

anxiety. *Psychol Med* 2015;**45**:2633–46. doi:10.1017/S0033291715000586

47 Forster S, Nunez Elizalde AO, Castle E, *et al.* Unraveling the Anxious Mind: Anxiety, Worry, and Frontal Engagement in Sustained Attention Versus Off-Task Processing. *Cereb Cortex N Y NY* 2015;**25**:609–18. doi:10.1093/cercor/bht248

Confidential: For Review Only

Table 1. Point prevalence of anxiety disorder in Chinese school children and adolescents.

	Identified Individuals	Unweighted point prevalence (%)	Unweighted 95% CI (%)	Adjusted Point Prevalence (%)	Adjusted 95% CI (%)
Generalized Anxiety Disorder	906	1.3	1.2-1.3	1.3	1.2-1.3
Social phobia	624	0.9	0.8-0.9	0.8	0.8-1.0
Separation anxiety disorder	416	0.6	0.5-0.6	0.6	0.5-1.0
Specific phobia	111	0.2	0.1-0.2	0.2	0.1-0.2
Panic disorder	167	0.2	0.2-0.3	0.2	0.2-0.2
Agoraphobia without panic	79	0.1	0.1-0.1	0.1	0.1-0.1

Confidential: For Review Only

Table 2. Differences in the prevalence of anxiety disorder in children and adolescents.

	Children(n=41332)			Adolescents(n=30775)			$\chi 2$	***P***
	Identified Individuals	Point prevalence (%)	95% CI	Identified Individuals	Point prevalence (%)	95% CI		
Panic disorder	51	0.12	0.09-0.16	116	0.38	0.31-0.45	30.91	**<0.001**
Generalized Anxiety Disorder	431	1.04	0.95-1.15	475	1.54	1.41-1.69	8.76	**0.003**
Specific Phobia	96	0.23	0.19-0.28	15	0.05	0.03-0.08	55.55	**<0.001**
Agoraphobia without panic	26	0.06	0．04-0.09	53	0.17	0.13-0.23	10.61	**0.001**
Social phobia	211	0.51	0．44-0.58	413	1.34	1.22-1.48	106.82	**<0.001**
Separation anxiety disorder	370	0.90	0.81-0.99	46	0.15	0.11-0.20	282.59	**<0.001**

Confidential: For Review Only

Table 3. Differences in the prevalence of anxiety disorder between boys and girls

	Boy(n=35953)			Girl(n=36154)			χ2	*P*
	Identified Individuals	Point prevalence (%)	95% CI	Identified Individuals	Point prevalence (%)	95% CI		
Panic disorder	53	0.15	0.11-0.19	114	0.32	0.26-0.38	14.61	**<0.001**
Generalized Anxiety Disorder	448	1.25	1.13-1.36	458	1.27	1.16-1.39	4.71	**0.02**
Specific Phobia	54	0.15	0.11-0.20	57	0.16	0.12-0.20	0.18	0.67
Agoraphobia without Panic	27	0.08	0.05-0.10	52	0.14	0.11-0.19	4.30	**0.03**
Social phobia	290	0.81	0.72-0.90	334	0.92	0.83-1.02	0.01	0.93
Separation anxiety disorder	201	0.56	0.48-0.64	215	0.59	0.52-0.68	0.78	0.38

Confidential: For Review Only

Table 4. Differences in the prevalence of anxiety disorder between developed and less developed areas.

	Developed areas (n=10549)			Less developed areas (n=63443)				
	Identified Individuals	Point prevalence (%)	95% CI	Identified Individuals	Point prevalence (%)	95% CI	**χ2**	***P***
Panic disorder	27	0.26	0.167-0. 37	140	0.22	0.19-0. 26	0.27	0.60
Generalized anxiety disorder	116	1.1	0.91-1.32	475	1.25	0.68-0.82	3.25	0.07
Specific phobia	19	0.18	0.11-0. 28	92	0.15	0.12-0.18	0.44	0.50
Agoraphobia without panic	12	0.11	0.06-0.20	67	0.11	0.08-0.13	$1.9*10^{-4}$	0.98
Social phobia	93	0.88	0.71-1.08	531	0.84	0.77-0.91	0.07	0.78
Separation anxiety disorder	67	0.64	0.49-0.81	349	0.55	0.49-0.62	0.90	0.34

Confidential: For Review Only

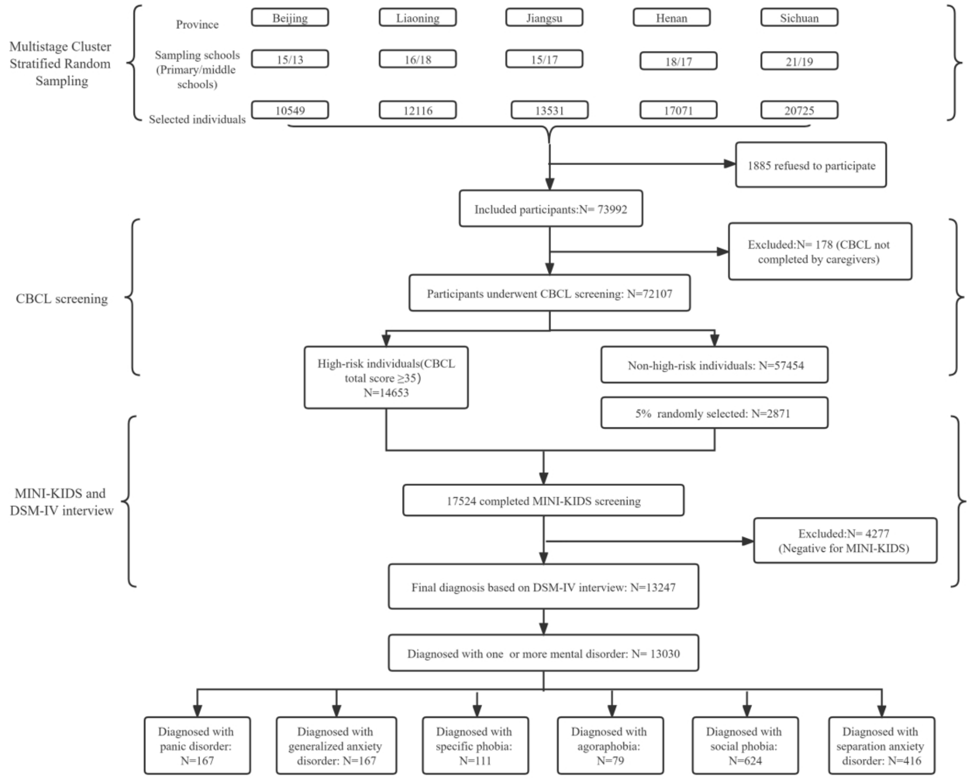

The flowchart for the included students

201x163mm (300 x 300 DPI)

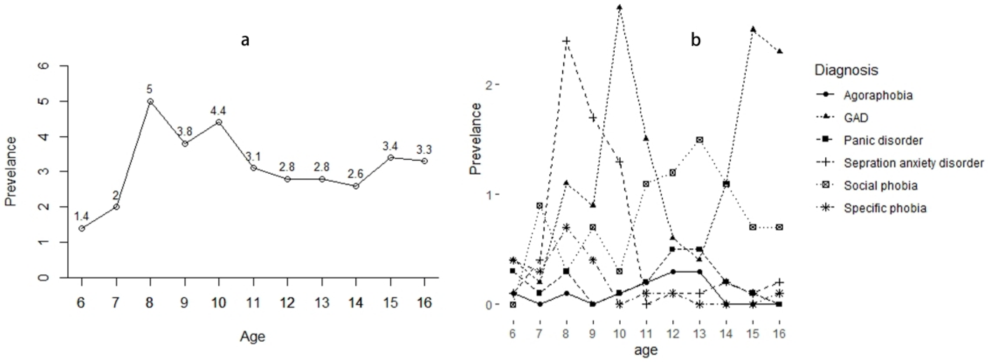

The prevalence of anxiety disorders change with the age

209x77mm (300 x 300 DPI)

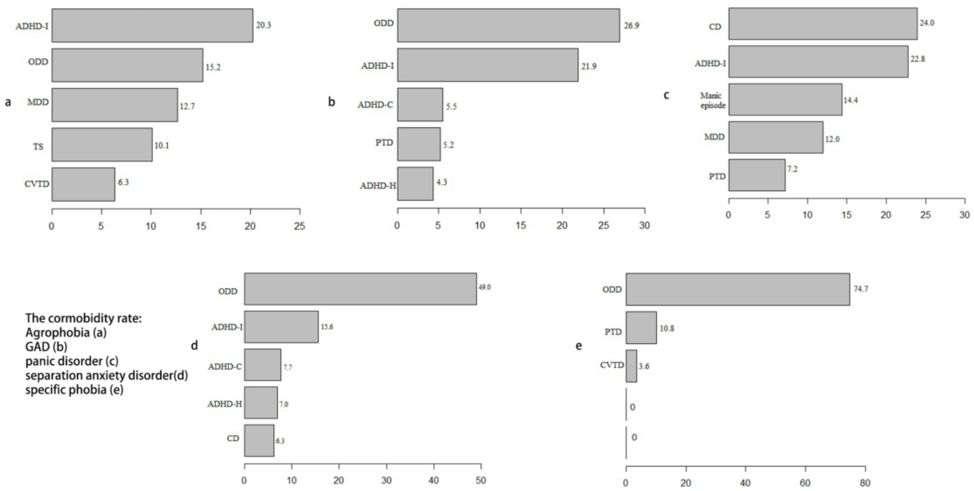

The comorbidity rate of anxiety disorders

209x107mm (300 x 300 DPI)

Supplementary table 1. List of the epidemiological studies of anxiety disorder in pediatric population.

Study	Screening and diagnosis tool	Sample source	Prevalence
Shen et al 2018	CBCL, MINI-KID and DSM-IV	School students aged 6 - 16 years from Hunan province (n= 17,071)	GAD: 1.77%, specific phobia: 0.74%, social phobia: 0.07%, separation anxiety disorder: 0.02%
Chen et al 2018	K-SADS	School students in Grade 3,5,7 in Taiwan (n=4816)	6-month prevalence:12.0% Life-time prevalence: 15.2%
Wagner et al 2017	SCOFF questionnaire and CDI-MD	Child aged 10-18 years enrolled from school and mental health institution in Austria (n=3615)	Point prevalence:10.17% Lifetime prevalence:15.58%
Qu et al 2015	CBCL, MINI-KID and DSM-IV	School students aged 6 - 16 years from Sichuan province (n= 20,752)	3.94%
Merikangas et al 2010	CIDI and DSM-IV	adolescents aged 13–18 including both community and school sample(n=10,123)	31.9%
Ulrike et al 2008	SDQ, SCARED	Child aged 7 to 17 years selected from local residents' registration system in German (n = 2863)	9.9% (decreased to 4.0% when considering impairment)
Leung et al 2008	DISC-IV	School students form Grade 7-9 in Hongkong (n= 541)	1-year prevalence: 30.2% (decreased to 6.9% when considering impairment)
Roberts et al 2007	DISC-IV and CGAS	Child aged 11–17 years selected from health maintenance organizations in Houston (n =4175)	1-year prevalence: 6.9%
Costello e al 2003	CAPA	Community sample aged 9-16 years in North Carolina (n=1420)	3-month prevalence:2.4%
Essau et al2000	DSM-IV	School students, aged 12-17 years in Bremen, German (n=1,035)	18.6%

Confidential: For Review Only

Footnote: CAPA: Child and adolescent psychiatric assessment; DISC-IV: Diagnostic Interview Schedule for Children-Version 4; K-SADS: Kiddie-Schedule for Affective Disorders and Schizophrenia; CGAS: Child Global Assessment Scale; CBCL: Child Behavior Checklist; MINI-KID: Mini-International Neuropsychiatric Interview for Children and Adolescents; CIDI: Composite International Diagnostic Interview; DSM-IV: Diagnostic and Statistical Manual of Mental Disorders—4th Edition; SDQ: strengths and difficulties questionnaire; SCARED: Screen for Child Anxiety Related Emotional Disorders; CDI-MD :Childrens' Diagnostic Interview for Mental Disorders

Confidential: For Review Only

BMJ Paediatrics Open

Prevalence and comorbidity of anxiety disorder in school-attending children and adolescents aged 6-16 in China

Journal:	*BMJ Paediatrics Open*
Manuscript ID	bmjpo-2023-001967.R1
Article Type:	Original research
Date Submitted by the Author:	20-Nov-2023
Complete List of Authors:	Wang, Fang; Beijing Children's Hospital Capital Medical University, Department of Psychiatry<br>Yang, Hanxue; Beijing Language and Culture University<br>Li, Fenghua; Chinese Academy of Sciences<br>Zheng, Yi; Capital Medical University, Department of pediatrics<br>Xu, Hui; National Center for Children's Health<br>Wang, Rui; Beijing Children's Hospital Capital Medical University<br>Li, Ying; Beijing Children's Hospital Capital Medical University, Department of Psychiatry<br>Cui, Yonghua; Beijing Children's Hospital Capital Medical University, Department of Psychiatry
Keywords:	Adolescent Health, Child Psychiatry, Epidemiology

SCHOLARONE™
Manuscripts

I, the Submitting Author has the right to grant and does grant on behalf of all authors of the Work (as defined in the below author licence), an exclusive licence and/or a non-exclusive licence for contributions from authors who are: i) UK Crown employees; ii) where BMJ has agreed a CC-BY licence shall apply, and/or iii) in accordance with the terms applicable for US Federal Government officers or employees acting as part of their official duties; on a worldwide, perpetual, irrevocable, royalty-free basis to BMJ Publishing Group Ltd ("BMJ") its licensees and where the relevant Journal is co-owned by BMJ to the co-owners of the Journal, to publish the Work in this journal and any other BMJ products and to exploit all rights, as set out in our licence.

The Submitting Author accepts and understands that any supply made under these terms is made by BMJ to the Submitting Author unless you are acting as an employee on behalf of your employer or a postgraduate student of an affiliated institution which is paying any applicable article publishing charge ("APC") for Open Access articles. Where the Submitting Author wishes to make the Work available on an Open Access basis (and intends to pay the relevant APC), the terms of reuse of such Open Access shall be governed by a Creative Commons licence – details of these licences and which Creative Commons licence will apply to this Work are set out in our licence referred to above.

Other than as permitted in any relevant BMJ Author's Self Archiving Policies, I confirm this Work has not been accepted for publication elsewhere, is not being considered for publication elsewhere and does not duplicate material already published. I confirm all authors consent to publication of this Work and authorise the granting of this licence.

Confidential: For Review Only

Prevalence and comorbidity of anxiety disorder in school-attending children and adolescents aged 6-16 in China

Fang Wang[1], Hanxue Yang[2], Fenghua Li[3], Yi Zheng[4], Hui Xu[5], Rui Wang [6], Ying Li[1*] Yonghua Cui[1*]

1, Department of Psychiatry, Beijing Children's Hospital, Capital Medical University, National Center for Children Healthy, Beijing, China.

2, School of Psychology, Beijing Language and Culture University, Beijing, China

3, Key Lab of Mental Health, Institute of Psychology, Chinese Academy of Sciences, Beijing, China

4, Department of pediatrics, Beijing Anding Hospital, Capital Medical University, Beijing, China

5, Big Data Center, Beijing Children's Hospital, Capital Medical University, National Center for Children's Health, Beijing, China

6, Laboratory of Nutrition and Development, Key Laboratory of Major Diseases in Children, Ministry of Education, Beijing Pediatric Research Institute, Beijing Children's Hospital, Capital Medical University, National Center for Children's Health, Beijing, China

Yonghua Cui, Beijing Children's Hospital, Capital Medical University, National Center for Children Healthy, 56 Nanlishi Road, Beijing 100101, China; Email: cuiyonghua@bch.com.cn; Tel/Fax number: +86(0)10-59616161

Ying Li, Beijing Children's Hospital, Capital Medical University, National Center for Children Healthy, 56 Nanlishi Road, Beijing 100101, China; Email: liying@bch.com.cn; Tel/Fax number: +86(0)10-59616161

Confidential: For Review Only

Abstract:

Background: The reported prevalence rate of anxiety disorder in the pediatric population varies widely between different counties. Currently, there is no national epidemiological survey of childhood anxiety disorder in China. This study aims to investigate the national prevalence of anxiety disorder, the distribution profiles of different subtypes and its comorbidity rates among school students.

Methods: A nationwide epidemiological survey of mental disorders in school students aged 6-16 years old was conducted. Participants were randomly recruited from five provinces in China. The Child Behavior Checklist was used to screen students at high risk for mental disorder. The final diagnosis was made based on the Diagnostic and Statistical Manual-IV. The point prevalence and comorbidity rate of anxiety disorder were calculated, and the difference between age, sex, and socioeconomic status groups was also compared.

Results: Generalized anxiety disorder (GAD) was the most common anxiety disorder in school-attending children and adolescents, with a prevalence rate of 1.3%, (95% CI: 1.2%-1.3%). Separation anxiety and specific phobia were more common in children than in adolescents. Girls had a higher prevalence of panic disorder (0.3% vs. 0.2%, $\chi^2 = 14.6$, $p < 0.001$) and agoraphobia (0.9% vs. 0.8%, $\chi^2 = 4.3$, $p = 0.03$) than boys. We found no significant difference between developed and less developed areas. Girls were more likely to have panic disorder and GAD than boys, with ratios of 2.13:1 and 1.01:1, respectively. The co-occurrence of anxiety disorder and attention-deficit and disruptive behavior disorder was very common, ranging from 40% to 85%.

Conclusions: Anxiety disorder was prevalent among school students in China and comorbidity with attention-deficit and disruptive disorder was very common. The data implie screening for anxiety disorder is needed in school settings. Policies should be adapted to provide psychological services to children and adolescents. Comprehensive assessment is recommended in clinical practice.

Keywords: anxiety disorder, prevalence, children and adolescents, comorbidity

Confidential: For Review Only

Key message:

- What is already known on this topic: Anxiety disorder is common in the pediatric population. It will cause great distress to both children and their families. However, as an internal symptom, anxiety maybe neglected by caregivers or teachers. This would prevent children and adolescents with anxiety disorders from accessing medical services. Currently, there is no nationwide epidemiological data on anxiety disorders in the pediatric population in China.
- What this study adds: This prevalence rate of anxiety disorder among school students in China was 4.7%. GAD was the most common subtype; the prevalence rate was 1.3%. Adolescents had a higher rates of panic disorder, GAD, agoraphobia, and social phobia than children. Girls had slightly higher rates of panic disorder, GAD and agoraphobia than boys. The comorbidity of attention-deficit and disruptive behavior disorder and anxiety disorder are very common. The prevalence of anxiety disorder was not influenced by the socioeconomic conditions.
- How this study might affect research, practice or policy: The data indicate screening for anxiety disorder maybe needed in school settings, and policies should be adapted to provide psychological services to children and adolescents.

Confidential: For Review Only

Confidential: For Review Only

1. Introduction

Anxiety disorders are characterized by excessive anxiety, worry, and related behaviors. Anxiety disorders, including panic disorder, generalized anxiety disorder (GAD), specific phobia, separation anxiety disorder, specific disorder and agoraphobia, and social phobia, are common in the pediatric population[1,2]. Comorbidity with other mental disorders, such as major depressive disorder and substance abuse, is very common in anxiety disorder[3]. Anxiety disorder and its comorbidities not only cause great distress to children, adolescents and their families but also have a negative impact on their educational achievement and social function in adulthood[3,4]. Furthermore, anxiety disorders increase the risk of other mental disorders[5]. Children with anxiety disorder are more likely to have depression disorder, suicidal behavior, and other psychiatric disorders in adulthood [6].As anxiety is an internal symptom, caregivers and teachers may not recognize the onset of anxiety in children and adolescents, which would prevent children and adolescents with anxiety disorders from accessing medical services[7]. It would therefore be of great importance to know the prevalence and characteristics of anxiety disorder in school settings in order to adapt our policies and provide psychological services to children and adolescents.

There have been many surveys exploring the prevalence of anxiety disorder in the pediatric population. However, the results vary widely between countries, ranging from 2% to 31%[3,8–11]. The prevalence of anxiety disorder was 6.3% in children aged 7-10 years and 4% in adolescents aged 11-17 years in a German national survey including 17641 participants[8]. A national survey in the United States including 10123 adolescents reported that the lifetime prevalence was as high as 31.9%[11]. Another recent study reported a lifetime prevalence of 15.6% among Austrian adolescents[10]. Comorbidity with other mental disorders was also very common in anxiety disorder, and the results were also inconsistent. The coexistence of different subtypes of anxiety disorder was 17-60%[9,12]. The comorbid rate of anxiety disorder and depression disorder was reported to be 10%-40%[13]. The co-occurrence rates of anxiety disorder and attention-deficit hyperactivity disorder (ADHD) was similar in different studies.

The comorbid rate was about 17-25%[9,14]. The prevalence of conduct disorder was also high in patients with anxiety disorder (18-38%)[9,15].

At present, the national prevalence rate of on anxiety disorders in the pediatric population in China is still not clear as there is no nationwide epidemiological survey. There are only few studies conducted locally. A survey conducted in Sichuan province, including 20752 6- to 16-year-old students, reported that the 6-month prevalence rate of anxiety disorder was 3.94%[16]. Chen et al. investigated 10118 children aged 7-12 years from primary and middle schools in Taiwan and reported that the lifetime prevalence of anxiety disorder was 15.2%[17]. The prevalence of any anxiety disorder among adolescents (Grade 7 to 9 high school students, the mean age were 13.8 ± 1.2 years) in Hong Kong was estimated to be 30.2%[18]. As you can see, the results vary a lot and cannot represent the overall situation in China. It is of great important to estimate the national prevalence rate of pediatric anxiety disorder as it will offer data support for government officials and mental health service providers. In this study, we further analyzed the data collected in the first national prevalence study of mental disorders in school-aged children and adolescents in China[19]. The aim was to investigate the prevalence of anxiety disorder subtypes, their comorbid conditions, and the distribution profile between different ages, gender, and locations.

2. Method

2.1 Included Participants

This is a two-stage survey carried out in primary and high schools in Beijing, Liaoning, Jiangsu, Sichuan, and Hunan Provinces. The age range of school students in is from 6 to 17 years. Due to the consideration of the College Entrance Examination, we decided not to include students in the fnal year of secondary school. So, the age range of students included in this survey was 6 to 16 years. The five provincial administrative regions were chosen by taking the geographical partition, economic development, and rural/urban factors in to consideration. In the official document, (National Bureau of Statistics of China, 2011), China was divided the country into northeastern, eastern, middle, and western regions geographically. Liaoning is chosen

Confidential: For Review Only

as the representative of northeast area and Jiangsu as the representative of eastern area, Sichuan as the representative of western area, Hunan as the representative of southern area. Beijing was picked as being representative of a Developed Urban Area, which had a gross domestic product per capita over 12,736 US dollars, and a population larger than 10 million. Then we randomly selected 2–4 prefecture divisions in these five areas. Fifteen prefecture divisions in total were selected finally. Next, in each prefectural division, simple sampling without replacement was used to select schools one by one (with a predetermined ratio of primary schools to middle schools as 1:1). Then, we randomly selected 2–5 classes in each grade of every school. The sample size was calculated using the formula $n = \frac{z^2_{1-\frac{\alpha}{2}}(1-p)}{p\varepsilon^2}$. We chose the prevalence of Tourette disorder (0.30%) to estimate the sample size as it was reported to be the lowest among children and adolescents aged 6–17[20]. with confidence coefficient of 95% (Z) relative error (ε) of 15%, 56742 participants would reach a statistic power of 1. We assumed 20% would be lost during the follow-up and decided to recruit 73992 participants. Based on this, we randomly chose 1764 classes from 169 schools (81 primary schools and 88 high schools). A more detailed description of the study design was described in our previous study[19,21].

2.2 CBCL screening

In the first stage, the Child Behavior Checklist (CBCL) was used for screening. The Chinese version of the CBCL has been shown to have good reliability and validity in clinical use[22]. And the internalizing syndrome subscale of CBCL had be proved to have good discriminant validity for anxiety disorder[23]. For all the enrolled students, their parents or other caregivers were asked to complete the CBCL, and the total problem score was calculated. According to the previous study of Liu et al., we chose a cut-off score of 35, which was the 90th percentile of the CBCL Total Problems score in their study[24]. Students with CBCL Total Problems scores higher than 35 (including 35) were identified as high-risk and underwent the second-stage interview.

2.3 MINI-KID and DSM-IV interview

This stage was performed approximately two weeks after the completion of the first stage. Considering the false-negative rate of CBCL screening, we also randomly selected 5% of the participants who showed CBCL-negative results and included them in the stage 2 assessment. They and the high-risk individuals underwent detailed interviews using the Mini-International Neuropsychiatric Interview for Children and Adolescents (MINI-KID). The MINI-KID is a widely used structured interview tool for the diagnosis of psychiatric disorders in children and adolescents[25]. In our study, the student and his or her parent or caregiver were interviewed at the same time. The MINI-KID assessment was carried out by psychiatrists with an attending physician title or higher, who were trained in the basic skill of MINI-KID. For estimation of the intraclass correlation coefficient (ICC) value, interview video clips of 10 children were watched and the children were rated. The ICC value was calculated based on the rating results of all the psychiatrists. The ICC value in our study was over 0.85. They also underwent the DSM-IV interview. Psychiatrists who performed the DSM-IV interview referred to the results of the MINI-KID rating, but the final diagnosis was made based on the DSM-IV criteria. The study procedure is illustrated in Figure 1.

Figure 1. Flowchart

2.4 Ethical approval

All subjects and their parents gave their informed consent for inclusion before they participated in the study. The study was conducted in accordance with the Declaration of Helsinki, and the protocol was approved by the Ethics Committee of the Anding Hospital, Capital Medical University (Project identification code:2012BAI01B02).

2.5 Statistical analysis

R software 3.4.3 was used for statistical analysis. The point prevalence of different subtypes of anxiety disorder and their comorbidity rates were calculated. The prevalence was adjusted based on the weights of their provincial region, prefectural division, county/district, school, and class. Taylor series linearization was used to calculate the 95% confidence intervals (CI) of the prevalence rate. The locations

Confidential: For Review Only

included in this study were classified into developed areas (Beijing) and developing areas (Liaoning, Jiangsu, Sichuan, and Hunan provinces) based on economic development conditions. The prevalence of anxiety disorder was also compared between age, gender, and socio-economic condition groups using Rao-Scott adjusted chi-square tests[26]. All statistical tests were two-sided tests, and $P < 0.05$ was considered significant.

3. Result

3.1. Characteristics of the included population

In total, 1764 classes from 81 primary schools and 88 middle schools (with 73992 students) were randomly selected. Finally, 72017 individuals completed the CBCL screening in stage 1, but 178 were excluded because their CBCL were completed by the teachers or themselves rather than by the caregivers. 17524 individuals (14653 high-risk individuals and 2871 randomly selected non-high-risk individuals) underwent stage 2 assessment. The mean age of the included population was 11.56 ± 5.12 years, and 66.2% lived in urban areas. The sex ratio of boys/girls was 0.99:1(36893/37099). Eighteen percent of the total sample were diagnosed with at least one mental disorder. Among those who were not at high risk, 5.5% were diagnosed with at least one mental disorder.

3.2 Point prevalence of anxiety disorder

The point prevalence of different subtypes of anxiety disorder and their 95% confidence intervals are shown in Table 1. The most prevalent anxiety disorder in children and adolescents was GAD, with an adjusted point prevalence of 1.3% (95% CI: 1.2%-1.3%). Followed by social phobia and separation anxiety disorder, the adjusted point prevalence was 0.8% (95% CI: 0.8% –1.0%) and 0.6% (95% CI: 0.5% – 1.0%), respectively. The adjusted point prevalence of panic disorder was 0.2% (95% CI: 0.2%-0.2%). A total of 0.2% (95% CI: 0.1%-0.2%) were diagnosed with specific phobia, and 0.1% (95% CI: 0.1%-0.1%) were diagnosed with agoraphobia.

Confidential: For Review Only

Table 1. Point prevalence of anxiety disorder in Chinese school children and adolescents.

	Identified Individuals	Unweighted point prevalence (%)	Unweighted 95% CI (%)	Adjusted Point Prevalence (%)	Adjusted 95% CI (%)
Generalized Anxiety Disorder	906	1.3	1.2-1.3	1.3	1.2-1.3
Social phobia	624	0.9	0.8-0.9	0.8	0.8-1.0
Separation anxiety disorder	416	0.6	0.5-0.6	0.6	0.5-1.0
Specific phobia	111	0.2	0.1-0.2	0.2	0.1-0.2
Panic disorder	167	0.2	0.2-0.3	0.2	0.2-0.2
Agoraphobia without panic	79	0.1	0.1-0.1	0.1	0.1-0.1

3.3 The effect of age, gender, and socio-economic conditions on the prevalence of anxiety disorder

The participants were divided into children and adolescents group based on the age. The age range of children was 6 to 12 years old, and the age range of adolescents was 13 to 16. As listed in Table 2, the distribution of anxiety disorder subtypes differed between different age groups. Adolescents had a higher rates of panic disorder, GAD, agoraphobia, and social phobia than children. Separation anxiety disorder and specific phobia were more common in children than in adolescents. The prevalence rate of separation anxiety was 0.90% (95% CI: 0.81%-0.99%) in children and 0.15% (95% CI: 0.11%-0.20%) in adolescents. ($\chi2 = 282.5, p < 0.001$). Specific phobia had a significantly higher rate in the child group. (0.23% vs 0.05%, $\chi2 = 55.5, p < 0.001$) As shown in Figure 2, age had a significant effect on the prevalence of different subtypes of anxiety disorder. The prevalence of anxiety disorder was relatively low before the age of 8 years old. For different subtypes of anxiety disorder, the peak prevalence age for separation anxiety disorder was between 7 and 10 years old, and the prevalence was relatively low after 11 years old. The prevalence of social phobia rises sharply after the

Confidential: For Review Only

age of 10 years old. The prevalence rate of anxiety disorder at different age is demonstrated in Supplementary File 1.

The prevalence of the panic disorder, GAD, and agoraphobia were significantly different between gender groups. The prevalence of panic disorder in girls was one time higher than in boys. In the group of boys, the prevalence rate was 0.15 % (95% CI: 0.11%-0.19%), while in the group of girls it was 0.32% (95% CI: 0.26%-0.38%, $\chi^2 = 14.6$, $p < 0.001$). Girls also had a slightly higher rates of GAD and agoraphobia than boys. The prevalence rate of agoraphobia was 0.08% (95% CI: 0.05%-0.10%) in the boy group and 0.14% (95% CI: 0.11%-0.19%) in the girl group. ($\chi^2 = 4.3$, p = 0.03). For GAD, the prevalence rate was 1.27% (95% CI: 1.16%-1.39%) in the girl group and 1.25% (95% CI: 1.13%-1.36%) in the boy group. ($\chi2 = 4.71$, p = 0.02). There were no significant differences in the prevalence of specific phobia, social phobia, and separation anxiety between boys and girls (for details, please see Table 3).

Based on the socioeconomic conditions, the five areas included in the study were divided into developed and underdeveloped areas. There was no significant difference in the prevalence of any anxiety disorder between these two groups. The results are listed in Table 4.

Table 2. Differences in the prevalence of anxiety disorder in children and adolescents.

	Children(n=41332)			Adolescents(n=30775)			***χ2***	***P***
	Identified Individuals	Point prevalence (%)	95% CI	Identified Individuals	Point prevalence (%)	95% CI		
Panic disorder	51	0.12	0.09-0.16	116	0.38	0.31-0.45	30.91	**<0.001**
Generalized Anxiety Disorder	431	1.04	0.95-1.15	475	1.54	1.41-1.69	8.76	**0.003**
Specific Phobia	96	0.23	0.19-0.28	15	0.05	0.03-0.08	55.55	**<0.001**

Confidential: For Review Only

Agoraphobia without panic	26	0.06	0．04-0.09	53	0.17	0.13-0.23	10.61	**0.001**
Social phobia	211	0.51	0．44-0.58	413	1.34	1.22-1.48	106.82	**<0.001**
Separation anxiety disorder	370	0.90	0.81-0.99	46	0.15	0.11-0.20	282.59	**<0.001**

Confidential: For Review Only

Table 3. Differences in the prevalence of anxiety disorder between boys and girls

	Boy(n=35953)			Girl(n=36154)			χ2	*P*
	Identified Individuals	Point prevalence (%)	95% CI	Identified Individuals	Point prevalence (%)	95% CI		
Panic disorder	53	0.15	0.11-0.19	114	0.32	0.26-0.38	14.61	**<0.001**
Generalized Anxiety Disorder	448	1.25	1.13-1.36	458	1.27	1.16-1.39	4.71	**0.02**
Specific Phobia	54	0.15	0.11-0.20	57	0.16	0.12-0.20	0.18	0.67
Agoraphobia without Panic	27	0.08	0.05-0.10	52	0.14	0.11-0.19	4.30	**0.03**
Social phobia	290	0.81	0.72-0.90	334	0.92	0.83-1.02	0.01	0.93
Separation anxiety disorder	201	0.56	0.48-0.64	215	0.59	0.52-0.68	0.78	0.38

Table 4. Differences in the prevalence of anxiety disorder between developed and less developed areas.

	Developed areas (n=10215)			Less developed areas (n=61892)			***χ2***	***P***
	Identified Individua	Point prevale	95% CI	Identified	Point prevalen	95% CI		

	ls	nce (%)		Individuals	ce (%)			
Panic disorder	27	0.26	0.167-0.37	140	0.23	0.19-0. 26	0.27	0.60
Generalized anxiety disorder	116	1.14	0.91-1.32	475	1.28	0.68-0.82	3.25	0.07
Specific phobia	19	0.19	0.11-0. 28	92	0.15	0.12-0.18	0.44	0.50
Agoraphobia without panic	12	0.12	0.06-0.20	67	0.11	0.08-0.13	$1.9*10^{-4}$	0.99
Social phobia	93	0.91	0.71-1.08	531	0.86	0.77-0.91	0.07	0.79
Separation anxiety disorder	67	0.66	0.49-0.81	349	0.56	0.49-0.62	0.90	0.34

Insert table 2-4 and figure 2

3.4 Comorbid conditions of anxiety disorder

A total of 70.3% (95% CI: 68.4%- 72.2%) of the children were diagnosed with anxiety disorder comorbid with at least one other mental disorder. A total of 42.2% (95% CI: 40.2%- 44.3%) had comorbidity with one other mental disorder, 27.5% (95% CI: 25.7%- 29.4%) were comorbid with two other mental disorders, and 0.6% (95% CI: 0.3%- 1.0%) were comorbid with three other mental disorders. The co-occurrence of anxiety disorder and ADHD, conduct disorder (CD) and oppositional defiant disorder (ODD) was very common. The most common comorbidity of GAD, specific phobia, and separation anxiety disorder was ODD, with comorbid rates of 26.9%, 74.7%, and 49%, respectively. The most common comorbidity for panic

Confidential: For Review Only

disorder was CD (24%). The ADHD-inattention subtype ranks as the most prevalent comorbidity for agoraphobia. Tic disorder was also common in anxiety disorder, and the comorbid rate of provisional tic disorder was 5.2% for GAD, 7.2% for panic disorder, and 10.8% for specific phobia. Tourette's syndrome and chronic vocal tic disorder were also common in agoraphobia. The concurrent rates were 10.1% and 6.3%, respectively. In our survey, there was no comorbidities of anxiety disorder and eating disorder, adjustment disorder, psychotic disorders, or pervasive developmental disorder (for details, please refer to figure 3).

Insert Figure 3

4. Discussion

This first national survey carried out in China showed that GAD was the most common anxiety disorder in school children and adolescents, with a prevalence rate of 1.3%, followed by social phobia (0.9%), separation anxiety disorder (0.6%), panic disorder (0.2%), specific phobia (0.2%), and agoraphobia (0.1%). The distribution of anxiety disorder was different among different age and gender group. Children had higher prevalence rate of separation anxiety disorder and specific phobia than adolescents. Girls were more likely to be affected by panic disorder and GAD than boys. As for comorbidities, attention-deficit and disruptive behavior disorders, tic disorders and depressive disorders were also commonly observed in the pediatric anxiety population.

Previous studies have shown that the prevalence of anxiety disorders in the pediatric population varies from 2% to 31% in different countries[8–11,16,17]. The prevalence of any anxiety disorder in our study was 4.7%. Our result was compatible with two other surveys carried out in Hunan and Sichuan provinces in China[9,16]. The prevalence rate was lower than that in many other studies[11,17,18]. The main reason might be related to the diagnostic criteria. In our survey, the DSM-IV criteria were used for the final diagnosis, which required functional impairment when making the diagnosis. A previous study showed that the prevalence of psychiatric disorders decreased sharply when the impairment criteria were applied[18]. In addition, stigma

Confidential: For Review Only

might be associated with low prevalence, as people in Asian countries are not used to reporting emotional problems[27]. Children and adolescents with self-stigma might not report their anxious experience[28]. As anxiety is an internal symptom, it might be ignored by caregivers.

Among the subtypes of anxiety disorder, GAD was the most prevalent anxiety disorder in our study (1.3%), which was consistent with two studies carried out in Hunan and Sichuan provinces, China. By investigating 17,071 primary and middle school students in Central Hunan Province, Shen et al reported that the 12-month prevalence of GAD was 1.77%, followed by 0.74% for a specific phobia, 0.07% for social phobia, and 0.02% for social anxiety disorder[9]. Roberts et al reported that agoraphobia was the most common anxiety disorder in their sample[29]. Several other studies reported that specific anxiety disorders had a much higher prevalence rate than other anxiety disorders[11,17]. However, the prevalence of panic disorder was relatively low (0.2%) in our study and other studies[11,16]. Notably, panic attacks are very common in the pediatric population. In the survey by Essau et al, 18% of adolescents reported experiencing panic attacks. However, only 0.5% met the diagnosis of panic disorder[3].

Both age and gender had effects on the prevalence of any anxiety disorder. Our results showed that girls were more likely to be affected by GAD and panic disorder. Gender differences have been reported in many previous studies[8,9,16]. Wagner et al reported that the prevalence rate of anxiety disorder was one time higher in girls than in boys (19.53 and 9.52%, respectively)[10]. However, some other studies found no gender difference[1,2]. The distribution of anxiety disorders was also different in different age groups. In general, adolescents had a higher rate of anxiety disorder, except for separation anxiety disorder. This was consistent with previous findings that anxiety disorder was less prevalent in younger children[11,30]. In our study, the peak age for separation anxiety disorder was between 7 and 10 years old. The finding was similar to that of Mohammadi et al[1]. This might be related to the fact that at this time, children separated from their parents and started attending school. Most previous studies have shown that young children have a higher prevalence rate of separation

Confidential: For Review Only

anxiety disorder[1,2]. In contrast, the prevalence of social anxiety disorder increases with age[30]. Our results found no difference between developed areas and less developed areas. The effect of socioeconomic factors on the prevalence of anxiety disorder is not clear. A cross-nation survey indicated that socioeconomic conditions might be associated with the differences in the prevalence of anxiety subtypes, and high-income countries had higher prevalence rates of social anxiety disorder than low- and lower-middle income countries[31]. Many other studies failed to find differences between different socioeconomic status groups[10,16].

In our study, 79.8% of children with anxiety disorder had at least one comorbidity. Previous studies indicated that the comorbid rate of anxiety disorder was high[32]. The comorbidity of other subtypes of anxiety disorder, depressive disorder, ADHD and disruptive, impulse control and conduct disorders was common[2,10,30]. Our results showed that the most prevalent comorbidities of anxiety disorder were disruptive, impulse control and conduct disorders. The most common comorbidity for GAD, specific phobia and separation anxiety disorder was ODD. CD ranked as the most common comorbidity for panic disorder. The high rate of comorbidity might be due to the following reasons. First, the symptoms of anxiety disorder and ODD and CD were highly associated[33]. Anger or irritability symptoms in the ODD lead to anxiety[34]. In addition, the functional impairment caused by ODD increased the risk of anxiety[35]. The concurrence of anxiety disorder and disruptive, impulse control and conduct disorders would cause more severe functional impairment for the individual [36]. Comorbidity with ADHD was also very common in our study. The comorbidity of anxiety disorder and ADHD could be explained by the attentional control theory[37]. Individuals in an anxious state have increased attention to threat-related stimuli and impaired attentional inhibition and shifting skills.[38]. Executive attention was impaired in children with anxiety disorders[39]. A neuroimaging study also confirmed deficits in prefrontal attentional control mechanisms in anxiety patients[40].

The study had several limitations. First, the participants were enrolled in schools. Children who did not attend school were not included, and some of them might may not attend school because of anxiety. This would undermine the representativeness of

Confidential: For Review Only

our sample. Second, the MINI-KID and the DSM-IV criteria were used for the diagnosis. However, the validity of the MINI-KID for the diagnosis of affective disorders, such as dysthymia, was not as good[25]. This may make the comorbidity results less comprehensive. The DSM-IV rather than DSM-5 diagnostic criteria was used in this study as it was started in October 2014, and the Chinese version of DSM-5 was published in July 2014. There was not enough time and resource to train clinicians to perform the DSM-5 assessment. The classification of anxiety disorder subtypes had changed a lot in DSM-5 criteria. For example, selective mutism was categorized as anxiety disorder in DSM-5 but not in DSM-IV. This would limit the comparability of the results with recent studies based on DSM-5. Thirdly, it should be noted that all diagnoses were made retrospectively, and recall bias could be a confounding factor for the results. Although according to the DSM-IV diagnostic criteria, functional impairment was considered while made the final diagnosis, there still lacks explicit measurement for functional impairment using psychological scales such as clinical global impression (CGI), this is another limitation of the study.

This study was a two-stage, national epidemiological survey using a stratified cluster sampling method. The participants represented school children and adolescents from different socio-economic backgrounds. The results demonstrated that anxiety disorder was common among school students. Girls were more likely to be affected by anxiety disorder than boys. The distribution of anxiety changed with age. Caregivers and teachers should pay more attention to the risk emotions of children. Our results also revealed that ADHD, ODD and CD often co-occurred with anxiety disorder. This implies that for children existing external symptoms such as aggressive symptoms and hyperactivity, the assessment of internal symptoms should not be neglected. A comprehensive assessment should be carried out in case the anxiety disorder is masked by external symptoms.

Funding statement: This work was supported by the National Natural Science Foundation of China (NSFC) under Grant No. 82001445, No. 82171538 and the Natural

Confidential: For Review Only

Science Foundation of Beijing Municipality under Grant No. 7212035, Beijing Hospitals Authority Youth Programme Grant No. QML20211203.

Competing interests: The authors declare no competing interests.

Patient and public involvement: Patients and/or the public were not involved in the design, or conduct, or reporting, or dissemination plans of this research.

Roles of authors: Yi Zheng and Yonghua Cui was responsible for the design and carry-out of the survey. Fang Wang contributed to the data analysis and writing of the manuscript. Hui Xu and Ying Li collaborated in the writing of the manuscript. Rui Wang, Fenghua Li and Ying Li participated in collecting data collection and digitalizing the data.

Confidential: For Review Only

Reference：

1. Mohammadi MR, Ahmadi N, Yazdi FR, et al. Prevalence, comorbidity and predictors of anxiety disorders among children and adolescents. Asian J Psychiatr 2020;53:102059; 10.1016/j.ajp.2020.102059.

2. Spence SH, Zubrick SR, Lawrence D. A profile of social, separation and generalized anxiety disorders in an Australian nationally representative sample of children and adolescents: Prevalence, comorbidity and correlates. Aust N Z J Psychiatry 2018;52(5):446–460; 10.1177/0004867417741981.

3. Essau CA, Conradt J, Petermann F. Frequency, comorbidity, and psychosocial impairment of anxiety disorders in German adolescents. J Anxiety Disord 2000;14(3):263–279; 10.1016/s0887-6185(99)00039-0.

4. Hossain B, Bent S, Hendren R. The association between anxiety and academic performance in children with reading disorder: A longitudinal cohort study. Dyslexia 2021;27(3):342–354; 10.1002/dys.1680.

5. Shek DTL, Chai W, Tan L. The relationship between anxiety and depression under the pandemic: The role of life meaning. Front Psychol 2022;13:1059330; 10.3389/fpsyg.2022.1059330.

6. Woodward LJ, Fergusson DM. Life course outcomes of young people with anxiety disorders in adolescence. J Am Acad Child Adolesc Psychiatry 2001;40(9):1086–1093; 10.1097/00004583-200109000-00018.

7. Richardson LP, Russo JE, Lozano P, et al. Factors associated with detection and receipt of treatment for youth with depression and anxiety disorders. Acad Pediatr 2010;10(1):36–40; 10.1016/j.acap.2009.09.011.

8. Ravens-Sieberer U, Wille N, Erhart M, et al. Prevalence of mental health problems among children and adolescents in Germany: results of the BELLA study within the National Health Interview and Examination Survey. Eur Child Adolesc Psychiatry 2008;17 Suppl 1:22–33; 10.1007/s00787-008-1003-2.

9. Shen Y-M, Chan BSM, Liu J-B, et al. The prevalence of psychiatric disorders among students aged 6~ 16 years old in central Hunan, China. BMC Psychiatry 2018;18:243; 10.1186/s12888-018-1823-7.

10. Wagner G, Zeiler M, Waldherr K, et al. Mental health problems in Austrian adolescents: a nationwide, two-stage epidemiological study applying DSM-5 criteria. European Child & Adolescent Psychiatry 2017;26(12):1483; 10.1007/s00787-017-0999-6.

11. Merikangas KR, He J-P, Burstein M, et al. Lifetime prevalence of mental disorders in U.S. adolescents: results from the National Comorbidity Survey Replication--Adolescent

Confidential: For Review Only

Supplement (NCS-A). J Am Acad Child Adolesc Psychiatry 2010;49(10):980–989; 10.1016/j.jaac.2010.05.017.

12. Walkup JT, Albano AM, Piacentini J, et al. Cognitive behavioral therapy, sertraline, or a combination in childhood anxiety. N Engl J Med 2008;359(26):2753–2766; 10.1056/NEJMoa0804633.

13. Choi EPH, Hui BPH, Wan EYF. Depression and Anxiety in Hong Kong during COVID-19. Int J Environ Res Public Health 2020;17(10):3740; 10.3390/ijerph17103740.

14. D'Agati E, Curatolo P, Mazzone L. Comorbidity between ADHD and anxiety disorders across the lifespan. Int J Psychiatry Clin Pract 2019;23(4):238–244; 10.1080/13651501.2019.1628277.

15. Ghandour RM, Sherman LJ, Vladutiu CJ, et al. Prevalence and Treatment of Depression, Anxiety, and Conduct Problems in US Children. J Pediatr 2019;206:256-267.e3; 10.1016/j.jpeds.2018.09.021.

16. Qu Y, Jiang H, Zhang N, et al. Prevalence of Mental Disorders in 6–16-Year-Old Students in Sichuan Province, China. Int J Environ Res Public Health 2015;12(5):5090–5107; 10.3390/ijerph120505090.

17. Chen Y-L, Chen WJ, Lin K-C, et al. Prevalence of DSM-5 mental disorders in a nationally representative sample of children in Taiwan: methodology and main findings. Epidemiol Psychiatr Sci 2019;29:e15; 10.1017/S2045796018000793.

18. Leung PWL, Hung S, Ho T, et al. Prevalence of DSM-IV disorders in Chinese adolescents and the effects of an impairment criterion: a pilot community study in Hong Kong. Eur Child Adolesc Psychiatry 2008;17(7):452–461; 10.1007/s00787-008-0687-7.

19. Li F, Cui Y, Li Y, et al. Prevalence of mental disorders in school children and adolescents in China: diagnostic data from detailed clinical assessments of 17,524 individuals. 2021;13.

20. Centers for Disease Control and Prevention (CDC). Prevalence of diagnosed Tourette syndrome in persons aged 6-17 years - United States, 2007. MMWR Morb Mortal Wkly Rep 2009;58(21):581–585.

21. Cui Y, Li F, Leckman JF, et al. The prevalence of behavioral and emotional problems among Chinese school children and adolescents aged 6-16: a national survey. Eur Child Adolesc Psychiatry 2021;30(2):233–241; 10.1007/s00787-020-01507-6.

22. Leung PWL, Kwong SL, Tang CP, et al. Test-retest reliability and criterion validity of the Chinese version of CBCL, TRF, and YSR. J Child Psychol Psychiatry 2006;47(9):970–973; 10.1111/j.1469-7610.2005.01570.x.

23. Seligman LD, Ollendick TH, Langley AK, et al. The utility of measures of child and adolescent anxiety: a meta-analytic review of the Revised Children's Manifest Anxiety Scale, the State-

Confidential: For Review Only

Trait Anxiety Inventory for Children, and the Child Behavior Checklist. J Clin Child Adolesc Psychol 2004;33(3):557–565; 10.1207/s15374424jccp3303_13.

24. Liu X, Kurita H, Guo C, et al. Prevalence and risk factors of behavioral and emotional problems among Chinese children aged 6 through 11 years. J Am Acad Child Adolesc Psychiatry 1999;38(6):708–715; 10.1097/00004583-199906000-00018.

25. Sheehan DV, Sheehan KH, Shytle RD, et al. Reliability and validity of the Mini International Neuropsychiatric Interview for Children and Adolescents (MINI-KID). J Clin Psychiatry 2010;71(3):313–326; 10.4088/JCP.09m05305whi.

26. Rao, J., & Scott, A. On simple adjustments to chi-square tests with sample survey data. The Annals of Statistics 1987.

27. Lynch H, McDonagh C, Hennessy E. Social Anxiety and Depression Stigma Among Adolescents. J Affect Disord 2021;281:744–750; 10.1016/j.jad.2020.11.073.

28. Calear AL, Batterham PJ, Torok M, et al. Help-seeking attitudes and intentions for generalised anxiety disorder in adolescents: the role of anxiety literacy and stigma. Eur Child Adolesc Psychiatry 2021;30(2):243–251; 10.1007/s00787-020-01512-9.

29. Roberts RE, Roberts CR, Xing Y. Rates of DSM-IV Psychiatric Disorders Among Adolescents in a Large Metropolitan Area. J Psychiatr Res 2007;41(11):959–967; 10.1016/j.jpsychires.2006.09.006.

30. Cummings CM, Caporino NE, Kendall PC. Comorbidity of anxiety and depression in children and adolescents: 20 years after. Psychol Bull 2014;140(3):816–845; 10.1037/a0034733.

31. Stein DJ, Lim CCW, Roest AM, et al. The cross-national epidemiology of social anxiety disorder: Data from the World Mental Health Survey Initiative. BMC Med 2017;15:143; 10.1186/s12916-017-0889-2.

32. Rapee RM, Lyneham HJ, Hudson JL, et al. Effect of comorbidity on treatment of anxious children and adolescents: results from a large, combined sample. J Am Acad Child Adolesc Psychiatry 2013;52(1):47–56; 10.1016/j.jaac.2012.10.002.

33. Humphreys KL, Aguirre VP, Lee SS. Association of anxiety and ODD/CD in children with and without ADHD. J Clin Child Adolesc Psychol 2012;41(3):370–377; 10.1080/15374416.2012.656557.

34. Drabick DAG, Gadow KD. Deconstructing oppositional defiant disorder: clinic-based evidence for an anger/irritability phenotype. J Am Acad Child Adolesc Psychiatry 2012;51(4):384–393; 10.1016/j.jaac.2012.01.010.

35. Evans SC, Burke JD, Roberts MC, et al. Irritability in child and adolescent psychopathology: An integrative review for ICD-11. Clin Psychol Rev 2017;53:29–45; 10.1016/j.cpr.2017.01.004.

Confidential: For Review Only

36. Martín V, Granero R, Ezpeleta L. Comorbidity of oppositional defiant disorder and anxiety disorders in preschoolers. Psicothema 2014;26(1):27–32; 10.7334/psicothema2013.75.

37. Eysenck MW, Derakshan N, Santos R, et al. Anxiety and cognitive performance: attentional control theory. Emotion 2007;7(2):336–353; 10.1037/1528-3542.7.2.336.

38. Ironside M, Browning M, Ansari TL, et al. Effect of Prefrontal Cortex Stimulation on Regulation of Amygdala Response to Threat in Individuals With Trait Anxiety. JAMA Psychiatry 2019;76(1):71–78; 10.1001/jamapsychiatry.2018.2172.

39. Mogg K, Salum GA, Bradley BP, et al. Attention network functioning in children with anxiety disorders, attention-deficit/hyperactivity disorder and non-clinical anxiety. Psychol Med 2015;45(12):2633–2646; 10.1017/S0033291715000586.

40. Forster S, Nunez Elizalde AO, Castle E, et al. Unraveling the Anxious Mind: Anxiety, Worry, and Frontal Engagement in Sustained Attention Versus Off-Task Processing. Cereb Cortex 2015;25(3):609–618; 10.1093/cercor/bht248.

Confidential: For Review Only

Figure legend

Figure 1. The flowchart of the study.

Figure 2. The prevalence rate of anxiety disorder at different age.

Figure 3. Comorbid conditions of anxiety disorder.

Table 1. The point prevalence of different subtypes of anxiety disorder.

Table 2. The prevalence rate of anxiety disorder in children and adolescents group.

Table 3. The prevalence rate of anxiety disorder in different gender group.

Table 4. The prevalence rate of anxiety disorder in developed and underdeveloped areas.

Supplementary file 1. The prevalence rate of anxiety disorder at different age.

Confidential: For Review Only

Table 1. Point prevalence of anxiety disorder in Chinese school children and adolescents.

	Identified Individuals	Unweighted point prevalence (%)	Unweighted 95% CI (%)	Adjusted Point Prevalence (%)	Adjusted 95% CI (%)
Generalized Anxiety Disorder	906	1.3	1.2-1.3	1.3	1.2-1.3
Social phobia	624	0.9	0.8-0.9	0.8	0.8-1.0
Separation anxiety disorder	416	0.6	0.5-0.6	0.6	0.5-1.0
Specific phobia	111	0.2	0.1-0.2	0.2	0.1-0.2
Panic disorder	167	0.2	0.2-0.3	0.2	0.2-0.2
Agoraphobia without panic	79	0.1	0.1-0.1	0.1	0.1-0.1

Confidential: For Review Only

Table 2. Differences in the prevalence of anxiety disorder in children and adolescents.

	Children(n=41332)			Adolescents(n=30775)			$\chi 2$	***P***
	Identified Individuals	Point prevalence (%)	95% CI	Identified Individuals	Point prevalence (%)	95% CI		
Panic disorder	51	0.12	0.09-0.16	116	0.38	0.31-0.45	30.91	**<0.001**
Generalized Anxiety Disorder	431	1.04	0.95-1.15	475	1.54	1.41-1.69	8.76	**0.003**
Specific Phobia	96	0.23	0.19-0.28	15	0.05	0.03-0.08	55.55	**<0.001**
Agoraphobia without panic	26	0.06	0．04-0.09	53	0.17	0.13-0.23	10.61	**0.001**
Social phobia	211	0.51	0．44-0.58	413	1.34	1.22-1.48	106.82	**<0.001**
Separation anxiety disorder	370	0.90	0.81-0.99	46	0.15	0.11-0.20	282.59	**<0.001**

Confidential: For Review Only

Table 3. Differences in the prevalence of anxiety disorder between boys and girls

	Boy(n=35953)			Girl(n=36154)			χ2	*P*
	Identified Individuals	Point prevalence (%)	95% CI	Identified Individuals	Point prevalence (%)	95% CI		
Panic disorder	53	0.15	0.11-0.19	114	0.32	0.26-0.38	14.61	**<0.001**
Generalized Anxiety Disorder	448	1.25	1.13-1.36	458	1.27	1.16-1.39	4.71	**0.02**
Specific Phobia	54	0.15	0.11-0.20	57	0.16	0.12-0.20	0.18	0.67
Agoraphobia without Panic	27	0.08	0.05-0.10	52	0.14	0.11-0.19	4.30	**0.03**
Social phobia	290	0.81	0.72-0.90	334	0.92	0.83-1.02	0.01	0.93
Separation anxiety disorder	201	0.56	0.48-0.64	215	0.59	0.52-0.68	0.78	0.38

Confidential: For Review Only

Table 4. Differences in the prevalence of anxiety disorder between developed and less developed areas.

	Developed areas (n=10215)			Less developed areas (n=61892)			*χ*2	*P*
	Identified Individuals	Point prevalence (%)	95% CI	Identified Individuals	Point prevalence (%)	95% CI		
Panic disorder	27	0.26	0.167-0. 37	140	0.23	0.19-0. 26	0.27	0.60
Generalized anxiety disorder	116	1.14	0.91-1.32	475	1.28	0.68-0.82	3.25	0.07
Specific phobia	19	0.19	0.11-0. 28	92	0.15	0.12-0.18	0.44	0.50
Agoraphobia without panic	12	0.12	0.06-0.20	67	0.11	0.08-0.13	$1.9*10^{-4}$	0.99
Social phobia	93	0.91	0.71-1.08	531	0.86	0.77-0.91	0.07	0.79
Separation anxiety disorder	67	0.66	0.49-0.81	349	0.56	0.49-0.62	0.90	0.34

Confidential: For Review Only

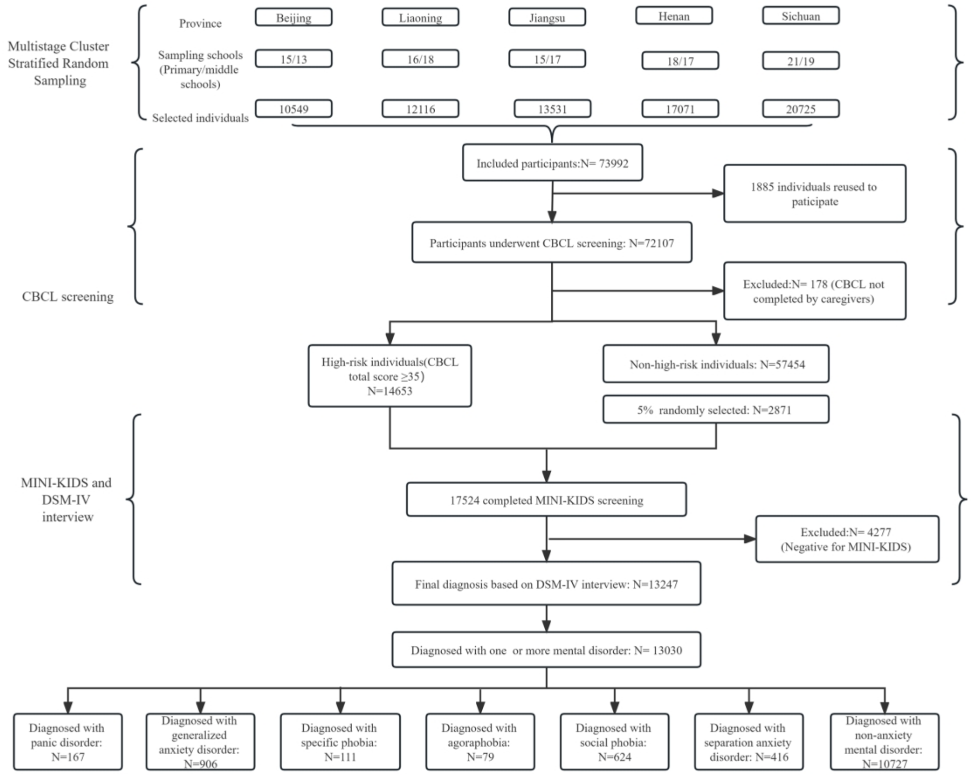

The flowchart to identify included participants

209x169mm (300 x 300 DPI)

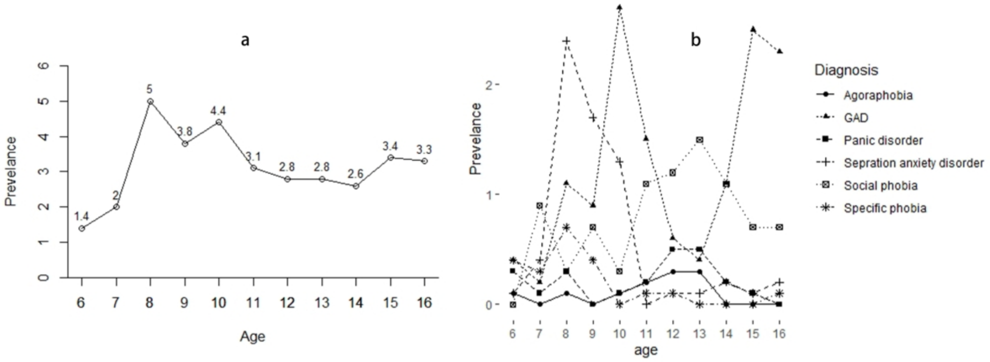

The prevalence of anxiety disorders change with the age

209x77mm (300 x 300 DPI)

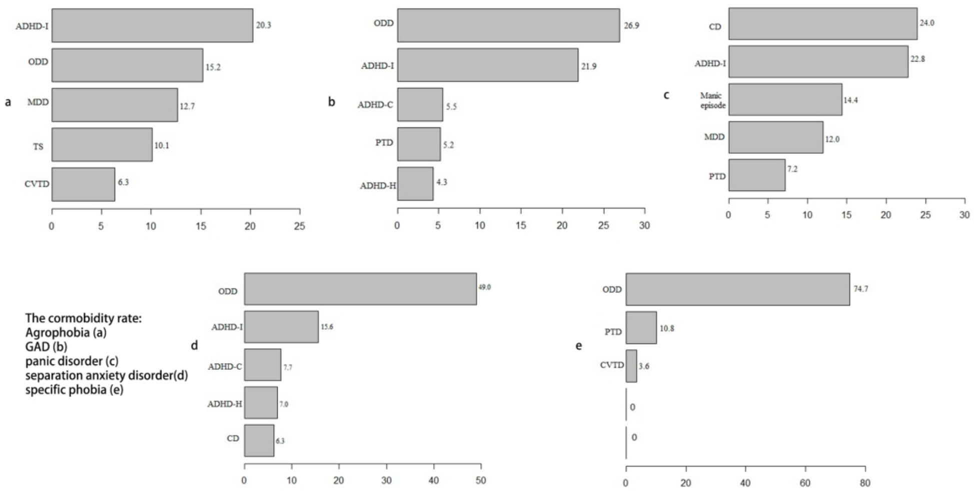

The comorbidity rate of anxiety disorders

209x107mm (300 x 300 DPI)

Supplementary File 1. The prevalence rate of anxiety disorder at different age.

Anxiety disorder \ Age	6	7	8	9	10	11	12	13	14	15	16
Panic disorder	0.3	0.1	0.3	0	0.1	0.2	0.5	0.5	0.2	0.1	0
Agoraphobia without panic	0.1	0	0.1	0	0.1	0.2	0.3	0.3	0	0	0
Separation anxiety disorder	0.1	0.4	2.4	1.7	1.3	0	0.1	0.1	0.2	0.1	0.1
Social phobia	0	0.9	0.3	0.7	0.3	1.1	1.2	1.5	1.1	0.7	0.5
Specific phobia	0.4	0.3	0.7	0.4	0	0.1	0.1	0	0	0	0.1
Generalized Anxiety Disorder	0.4	0.2	1.1	0.9	2.7	1.5	0.6	0.4	1.1	2.5	2.2
Total	1.4	2.0	5.0	3.8	4.4	3.1	2.8	2.8	2.6	3.4	3.0

Confidential: For Review Only

BMJ Paediatrics Open

Prevalence and comorbidity of anxiety disorder in school-attending children and adolescents aged 6-16 in China

Journal:	*BMJ Paediatrics Open*
Manuscript ID	bmjpo-2023-001967.R2
Article Type:	Original research
Date Submitted by the Author:	27-Dec-2023
Complete List of Authors:	Wang, Fang; Beijing Children's Hospital Capital Medical University, Department of Psychiatry<br>Yang, Hanxue; Beijing Language and Culture University<br>Li, Fenghua; Chinese Academy of Sciences<br>Zheng, Yi; Capital Medical University, Department of pediatrics<br>Xu, Hui; National Center for Children's Health<br>Wang, Rui; Beijing Children's Hospital Capital Medical University<br>Li, Ying; Beijing Children's Hospital Capital Medical University, Department of Psychiatry<br>Cui, Yonghua; Beijing Children's Hospital Capital Medical University, Department of Psychiatry
Keywords:	Adolescent Health, Child Psychiatry, Epidemiology

SCHOLARONE™
Manuscripts

I, the Submitting Author has the right to grant and does grant on behalf of all authors of the Work (as defined in the below author licence), an exclusive licence and/or a non-exclusive licence for contributions from authors who are: i) UK Crown employees; ii) where BMJ has agreed a CC-BY licence shall apply, and/or iii) in accordance with the terms applicable for US Federal Government officers or employees acting as part of their official duties; on a worldwide, perpetual, irrevocable, royalty-free basis to BMJ Publishing Group Ltd ("BMJ") its licensees and where the relevant Journal is co-owned by BMJ to the co-owners of the Journal, to publish the Work in this journal and any other BMJ products and to exploit all rights, as set out in our licence.

The Submitting Author accepts and understands that any supply made under these terms is made by BMJ to the Submitting Author unless you are acting as an employee on behalf of your employer or a postgraduate student of an affiliated institution which is paying any applicable article publishing charge ("APC") for Open Access articles. Where the Submitting Author wishes to make the Work available on an Open Access basis (and intends to pay the relevant APC), the terms of reuse of such Open Access shall be governed by a Creative Commons licence – details of these licences and which Creative Commons licence will apply to this Work are set out in our licence referred to above.

Other than as permitted in any relevant BMJ Author's Self Archiving Policies, I confirm this Work has not been accepted for publication elsewhere, is not being considered for publication elsewhere and does not duplicate material already published. I confirm all authors consent to publication of this Work and authorise the granting of this licence.

Confidential: For Review Only

Prevalence and comorbidity of anxiety disorder in school-attending children and adolescents aged 6-16 in China

Fang Wang[1], Hanxue Yang[2], Fenghua Li[3], Yi Zheng[4], Hui Xu[5], Rui Wang [6], Ying Li[1*] Yonghua Cui[1*]

1, Department of Psychiatry, Beijing Children's Hospital, Capital Medical University, National Center for Children Healthy, Beijing, China.

2, School of Psychology, Beijing Language and Culture University, Beijing, China

3, Key Lab of Mental Health, Institute of Psychology, Chinese Academy of Sciences, Beijing, China

4, Department of pediatrics, Beijing Anding Hospital, Capital Medical University, Beijing, China

5, Big Data Center, Beijing Children's Hospital, Capital Medical University, National Center for Children's Health, Beijing, China

6, Laboratory of Nutrition and Development, Key Laboratory of Major Diseases in Children, Ministry of Education, Beijing Pediatric Research Institute, Beijing Children's Hospital, Capital Medical University, National Center for Children's Health, Beijing, China

Yonghua Cui, Beijing Children's Hospital, Capital Medical University, National Center for Children Healthy, 56 Nanlishi Road, Beijing 100101, China; Email: cuiyonghua@bch.com.cn; Tel/Fax number: +86(0)10-59616161

Ying Li, Beijing Children's Hospital, Capital Medical University, National Center for Children Healthy, 56 Nanlishi Road, Beijing 100101, China; Email: liying@bch.com.cn; Tel/Fax number: +86(0)10-59616161

Confidential: For Review Only

Abstract:

Background: The reported prevalence rate of anxiety disorder in the pediatric population varies widely between different counties. Currently, there is no national epidemiological survey of childhood anxiety disorder in China. This study aims to investigate the national prevalence of anxiety disorder, the distribution profiles of different subtypes and its comorbidity rates among school students.

Methods: A nationwide epidemiological survey of mental disorders in school students aged 6-16 years old was conducted. Participants were randomly recruited from five provinces in China. The Child Behavior Checklist was used to screen students at high risk for mental disorder. The final diagnosis was made based on the Diagnostic and Statistical Manual-IV. The point prevalence and comorbidity rate of anxiety disorder were calculated, and the difference between age, sex, and socioeconomic status groups was also compared.

Results: Generalized anxiety disorder (GAD) was the most common anxiety disorder in school-attending children and adolescents, with a prevalence rate of 1.3%, (95% CI: 1.2%-1.3%). Separation anxiety and specific phobia were more common in children than in adolescents. Girls had a higher prevalence of panic disorder (0.3% vs. 0.2%, $\chi^2 = 14.6$, $p < 0.001$) and agoraphobia (0.9% vs. 0.8%, $\chi^2 = 4.3$, $p = 0.03$) than boys. We found no significant difference between developed and less developed areas. Girls were more likely to have panic disorder and GAD than boys, with ratios of 2.13:1 and 1.01:1, respectively. The co-occurrence of anxiety disorder and attention-deficit and disruptive behavior disorder was very common, ranging from 40% to 85%.

Conclusions: Anxiety disorder was prevalent among school students in China and comorbidity with attention-deficit and disruptive disorder was very common. The data implie screening for anxiety disorder is needed in school settings. Policies should be adapted to provide psychological services to children and adolescents. Comprehensive assessment is recommended in clinical practice.

Keywords: anxiety disorder, prevalence, children and adolescents, comorbidity

Confidential: For Review Only

Key message:

- What is already known on this topic: Anxiety disorder is common in the pediatric population. It will cause great distress to both children and their families. However, as an internal symptom, anxiety maybe neglected by caregivers or teachers. This would prevent children and adolescents with anxiety disorders from accessing medical services. Currently, there is no nationwide epidemiological data on anxiety disorders in the pediatric population in China.

- What this study adds: This prevalence rate of anxiety disorder among school students in China was 4.7%. GAD was the most common subtype; the prevalence rate was 1.3%. Adolescents had a higher rates of panic disorder, GAD, agoraphobia, and social phobia than children. Girls had slightly higher rates of panic disorder, GAD and agoraphobia than boys. The comorbidity of attention-deficit and disruptive behavior disorder and anxiety disorder are very common. The prevalence of anxiety disorder was not influenced by the socioeconomic conditions.

- How this study might affect research, practice or policy: The data indicate screening for anxiety disorder maybe needed in school settings, and policies should be adapted to provide psychological services to children and adolescents.

Confidential: For Review Only

1. Introduction

Anxiety disorders are characterized by excessive anxiety, worry, and related behaviors. Anxiety disorders, including panic disorder, generalized anxiety disorder (GAD), specific phobia, separation anxiety disorder, specific disorder and agoraphobia, and social phobia, are common in the pediatric population[1,2]. Comorbidity with other mental disorders, such as major depressive disorder and substance abuse, is very common in anxiety disorder[3]. Anxiety disorder and its comorbidities not only cause great distress to children, adolescents and their families but also have a negative impact on their educational achievement and social function in adulthood[3,4]. Furthermore, anxiety disorders increase the risk of other mental disorders[5]. Children with anxiety disorder are more likely to have depression disorder, suicidal behavior, and other psychiatric disorders in adulthood [6].As anxiety is an internal symptom, caregivers and teachers may not recognize the onset of anxiety in children and adolescents, which would prevent children and adolescents with anxiety disorders from accessing medical services[7]. It would therefore be of great importance to know the prevalence and characteristics of anxiety disorder in school settings in order to adapt our policies and provide psychological services to children and adolescents.

There have been many surveys exploring the prevalence of anxiety disorder in the pediatric population. However, the results vary widely between countries, ranging from 2% to 31%[3,8–11]. The prevalence of anxiety disorder was 6.3% in children aged 7-10 years and 4% in adolescents aged 11-17 years in a German national survey including 17641 participants[8]. A national survey in the United States including 10123 adolescents reported that the lifetime prevalence was as high as 31.9%[11]. Another recent study reported a lifetime prevalence of 15.6% among Austrian adolescents[10]. Comorbidity with other mental disorders was also very common in anxiety disorder, and the results were also inconsistent. The coexistence of different subtypes of anxiety disorder was 17-60%[9,12]. The comorbid rate of anxiety disorder and depression disorder was reported to be 10%-40%[13]. The co-occurrence rates of anxiety disorder and attention-deficit hyperactivity disorder (ADHD) was similar in different studies.

Confidential: For Review Only

The comorbid rate was about 17-25%[9,14]. The prevalence of conduct disorder was also high in patients with anxiety disorder (18-38%)[9,15].

At present, the national prevalence rate of on anxiety disorders in the pediatric population in China is still not clear as there is no nationwide epidemiological survey. There are only few studies conducted locally. A survey conducted in Sichuan province, including 20752 6- to 16-year-old students, reported that the 6-month prevalence rate of anxiety disorder was 3.94%[16]. Chen et al. investigated 10118 children aged 7-12 years from primary and middle schools in Taiwan and reported that the lifetime prevalence of anxiety disorder was 15.2%[17]. The prevalence of any anxiety disorder among adolescents (Grade 7 to 9 high school students, the mean age were 13.8 ± 1.2 years) in Hong Kong was estimated to be 30.2%[18]. As you can see, the results vary a lot and cannot represent the overall situation in China. It is of great important to estimate the national prevalence rate of pediatric anxiety disorder as it will offer data support for government officials and mental health service providers. In this study, we further analyzed the data collected in the first national prevalence study of mental disorders in school-aged children and adolescents in China[19]. The aim was to investigate the prevalence of anxiety disorder subtypes, their comorbid conditions, and the distribution profile between different ages, gender, and locations.

2. Method

2.1 Included Participants

This is a two-stage survey carried out in primary and high schools in Beijing, Liaoning, Jiangsu, Sichuan, and Hunan Provinces. The age range of school students in is from 6 to 17 years. Due to the consideration of the College Entrance Examination, we decided not to include students in the fnal year of secondary school. So, the age range of students included in this survey was 6 to 16 years. The five provincial administrative regions were chosen by taking the geographical partition, economic development, and rural/urban factors in to consideration. In the official document, (National Bureau of Statistics of China, 2011), China was divided the country into northeastern, eastern, middle, and western regions geographically. Liaoning is chosen

as the representative of northeast area and Jiangsu as the representative of eastern area, Sichuan as the representative of western area, Hunan as the representative of southern area. Beijing was picked as being representative of a Developed Urban Area, which had a gross domestic product per capita over 12,736 US dollars, and a population larger than 10 million. Then we randomly selected 2–4 prefecture divisions in these five areas. Fifteen prefecture divisions in total were selected finally. Next, in each prefectural division, simple sampling without replacement was used to select schools one by one (with a predetermined ratio of primary schools to middle schools as 1:1). Then, we randomly selected 2–5 classes in each grade of every school. The sample size was calculated using the formula $n = \frac{z^2_{1-\frac{\alpha}{2}}(1-p)}{p\varepsilon^2}$ [19,20].We chose the prevalence of Tourette disorder (0.30%) to estimate the sample size as it was reported to be the lowest among children and adolescents aged 6–17[21], with confidence coefficient of 95% (Z) relative error (ε) of 15%, 56742 participants would reach a statistic power of 1. We assumed 20% would be lost during the follow-up and decided to recruit 73992 participants. Based on this, we randomly chose 1764 classes from 169 schools (81 primary schools and 88 high schools). A more detailed description of the study design was described in our previous study[19].

2.2 CBCL screening

In the first stage, the Child Behavior Checklist (CBCL) was used for screening. The Chinese version of the CBCL has been shown to have good reliability and validity in clinical use[22]. And the internalizing syndrome subscale of CBCL had be proved to have good discriminant validity for anxiety disorder[23]. For all the enrolled students, their parents or other caregivers were asked to complete the CBCL, and the total problem score was calculated. According to the previous study of Liu et al., we chose a cut-off score of 35, which was the 90th percentile of the CBCL Total Problems score in their study[24]. Students with CBCL Total Problems scores higher than 35 (including 35) were identified as high-risk and underwent the second-stage interview.

2.3 MINI-KID and DSM-IV interview

This stage was performed approximately two weeks after the completion of the first stage. Considering the false-negative rate of CBCL screening, we also randomly selected 5% of the participants who showed CBCL-negative results and included them in the stage 2 assessment. They and the high-risk individuals underwent detailed interviews using the Mini-International Neuropsychiatric Interview for Children and Adolescents (MINI-KID). The MINI-KID is a widely used structured interview tool for the diagnosis of psychiatric disorders in children and adolescents[25]. In our study, the student and his or her parent or caregiver were interviewed at the same time. The MINI-KID assessment was carried out by psychiatrists with an attending physician title or higher, who were trained in the basic skill of MINI-KID. For estimation of the intraclass correlation coefficient (ICC) value, interview video clips of 10 children were watched and the children were rated. The ICC value was calculated based on the rating results of all the psychiatrists. The ICC value in our study was over 0.85. They also underwent the DSM-IV interview. Psychiatrists who performed the DSM-IV interview referred to the results of the MINI-KID rating, but the final diagnosis was made based on the DSM-IV criteria. The study procedure is illustrated in Figure 1.

Figure 1. Flowchart

2.4 Ethical approval

All subjects and their parents gave their informed consent for inclusion before they participated in the study. The study was conducted in accordance with the Declaration of Helsinki, and the protocol was approved by the Ethics Committee of the Anding Hospital, Capital Medical University (Project identification code:2012BAI01B02).

2.5 Statistical analysis

R software 3.4.3 was used for statistical analysis. The point prevalence of different subtypes of anxiety disorder and their comorbidity rates were calculated. The prevalence was adjusted based on the weights of their provincial region, prefectural division, county/district, school, and class. Taylor series linearization was used to calculate the 95% confidence intervals (CI) of the prevalence rate. The locations

Confidential: For Review Only

included in this study were classified into developed areas (Beijing) and developing areas (Liaoning, Jiangsu, Sichuan, and Hunan provinces) based on economic development conditions. The prevalence of anxiety disorder was also compared between age, gender, and socio-economic condition groups using Rao-Scott adjusted chi-square tests[26]. All statistical tests were two-sided tests, and $P < 0.05$ was considered significant.

3. Result

3.1. Characteristics of the included population

In total, 1764 classes from 81 primary schools and 88 middle schools (with 73992 students) were randomly selected. Finally, 72017 individuals completed the CBCL screening in stage 1, but 178 were excluded because their CBCL were completed by the teachers or themselves rather than by the caregivers. 17524 individuals (14653 high-risk individuals and 2871 randomly selected non-high-risk individuals) underwent stage 2 assessment. The mean age of the included population was 11.56 ± 5.12 years, and 66.2% lived in urban areas. The sex ratio of boys/girls was 0.99:1(36893/37099). Eighteen percent of the total sample were diagnosed with at least one mental disorder. Among those who were not at high risk, 5.5% were diagnosed with at least one mental disorder.

3.2 Point prevalence of anxiety disorder

The point prevalence of different subtypes of anxiety disorder and their 95% confidence intervals are shown in Table 1. The most prevalent anxiety disorder in children and adolescents was GAD, with an adjusted point prevalence of 1.3% (95% CI: 1.2%-1.3%). Followed by social phobia and separation anxiety disorder, the adjusted point prevalence was 0.8% (95% CI: 0.8% –1.0%) and 0.6% (95% CI: 0.5% – 1.0%), respectively. The adjusted point prevalence of panic disorder was 0.2% (95% CI: 0.2%-0.2%). A total of 0.2% (95% CI: 0.1%-0.2%) were diagnosed with specific phobia, and 0.1% (95% CI: 0.1%-0.1%) were diagnosed with agoraphobia.

Table 1. Point prevalence of anxiety disorder in Chinese school children and adolescents.

	Identified Individuals	Unweighted point prevalence (%)	Unweighted 95% CI (%)	Adjusted Point Prevalence (%)	Adjusted 95% CI (%)
Generalized Anxiety Disorder	906	1.3	1.2-1.3	1.3	1.2-1.3
Social phobia	624	0.9	0.8-0.9	0.8	0.8-1.0
Separation anxiety disorder	416	0.6	0.5-0.6	0.6	0.5-1.0
Specific phobia	111	0.2	0.1-0.2	0.2	0.1-0.2
Panic disorder	167	0.2	0.2-0.3	0.2	0.2-0.2
Agoraphobia without panic	79	0.1	0.1-0.1	0.1	0.1-0.1

3.3 The effect of age, gender, and socio-economic conditions on the prevalence of anxiety disorder

The participants were divided into children and adolescents group based on the age. The age range of children was 6 to 12 years old, and the age range of adolescents was 13 to 16. As listed in Table 2, the distribution of anxiety disorder subtypes differed between different age groups. Adolescents had a higher rates of panic disorder, GAD, agoraphobia, and social phobia than children. Separation anxiety disorder and specific phobia were more common in children than in adolescents. The prevalence rate of separation anxiety was 0.90% (95% CI: 0.81%-0.99%) in children and 0.15% (95% CI: 0.11%-0.20%) in adolescents. ($\chi 2 = 282.5, p < 0.001$). Specific phobia had a significantly higher rate in the child group. (0.23% vs 0.05%, $\chi 2 = 55.5, p < 0.001$) As shown in Figure 2, age had a significant effect on the prevalence of different subtypes of anxiety disorder. The prevalence of anxiety disorder was relatively low before the age of 8 years old. For different subtypes of anxiety disorder, the peak prevalence age for separation anxiety disorder was between 7 and 10 years old, and the prevalence was relatively low after 11 years old. The prevalence of social phobia rises sharply after the

Confidential: For Review Only

age of 10 years old. The prevalence rate of anxiety disorder at different age is demonstrated in Supplementary File 1.

The prevalence of the panic disorder, GAD, and agoraphobia were significantly different between gender groups. The prevalence of panic disorder in girls was one time higher than in boys. In the group of boys, the prevalence rate was 0.15 % (95% CI: 0.11%-0.19%), while in the group of girls it was 0.32% (95% CI: 0.26%-0.38%, $\chi^2 = 14.6$, $p < 0.001$). Girls also had a slightly higher rates of GAD and agoraphobia than boys. The prevalence rate of agoraphobia was 0.08% (95% CI: 0.05%-0.10%) in the boy group and 0.14% (95% CI: 0.11%-0.19%) in the girl group. ($\chi^2 = 4.3$, p = 0.03). For GAD, the prevalence rate was 1.27% (95% CI: 1.16%-1.39%) in the girl group and 1.25% (95% CI: 1.13%-1.36%) in the boy group. ($\chi 2 = 4.71$, p = 0.02). There were no significant differences in the prevalence of specific phobia, social phobia, and separation anxiety between boys and girls (for details, please see Table 3).

Based on the socioeconomic conditions, the five areas included in the study were divided into developed and underdeveloped areas. There was no significant difference in the prevalence of any anxiety disorder between these two groups. The results are listed in Table 4.

Table 2. Differences in the prevalence of anxiety disorder in children and adolescents.

	Children(n=41332)			Adolescents(n=30775)			***χ2***	
	Identified Individuals	Point prevalence (%)	95% CI	Identified Individuals	Point prevalence (%)	95% CI		***P***
Panic disorder	51	0.12	0.09-0.16	116	0.38	0.31-0.45	30.91	**<0.001**
Generalized Anxiety Disorder	431	1.04	0.95-1.15	475	1.54	1.41-1.69	8.76	**0.003**
Specific Phobia	96	0.23	0.19-0.28	15	0.05	0.03-0.08	55.55	**<0.001**

Confidential: For Review Only

Agoraphobia without panic	26	0.06	0．04-0.09	53	0.17	0.13-0.23	10.61	**0.001**
Social phobia	211	0.51	0．44-0.58	413	1.34	1.22-1.48	106.82	**<0.001**
Separation anxiety disorder	370	0.90	0.81-0.99	46	0.15	0.11-0.20	282.59	**<0.001**

Confidential: For Review Only

Table 3. Differences in the prevalence of anxiety disorder between boys and girls

	Boy(n=35953)			Girl(n=36154)			χ2	*P*
	Identified Individuals	Point prevalence (%)	95% CI	Identified Individuals	Point prevalence (%)	95% CI		
Panic disorder	53	0.15	0.11-0.19	114	0.32	0.26-0.38	14.61	**<0.001**
Generalized Anxiety Disorder	448	1.25	1.13-1.36	458	1.27	1.16-1.39	4.71	**0.02**
Specific Phobia	54	0.15	0.11-0.20	57	0.16	0.12-0.20	0.18	0.67
Agoraphobia without Panic	27	0.08	0.05-0.10	52	0.14	0.11-0.19	4.30	**0.03**
Social phobia	290	0.81	0.72-0.90	334	0.92	0.83-1.02	0.01	0.93
Separation anxiety disorder	201	0.56	0.48-0.64	215	0.59	0.52-0.68	0.78	0.38

Table 4. Differences in the prevalence of anxiety disorder between developed and less developed areas.

	Developed areas (n=10215)			Less developed areas (n=61892)			***χ2***	***P***
	Identified Individua	Point prevale	95% CI	Identified	Point prevalen	95% CI		

	ls	nce (%)		Individuals	ce (%)			
Panic disorder	27	0.26	0.167-0.37	140	0.23	0.19-0. 26	0.27	0.60
Generalized anxiety disorder	116	1.14	0.91-1.32	475	1.28	0.68-0.82	3.25	0.07
Specific phobia	19	0.19	0.11-0. 28	92	0.15	0.12-0.18	0.44	0.50
Agoraphobia without panic	12	0.12	0.06-0.20	67	0.11	0.08-0.13	$1.9*10^{-4}$	0.99
Social phobia	93	0.91	0.71-1.08	531	0.86	0.77-0.91	0.07	0.79
Separation anxiety disorder	67	0.66	0.49-0.81	349	0.56	0.49-0.62	0.90	0.34

Insert table 2-4 and figure 2

3.4 Comorbid conditions of anxiety disorder

A total of 70.3% (95% CI: 68.4%- 72.2%) of the children were diagnosed with anxiety disorder comorbid with at least one other mental disorder. A total of 42.2% (95% CI: 40.2%- 44.3%) had comorbidity with one other mental disorder, 27.5% (95% CI: 25.7%- 29.4%) were comorbid with two other mental disorders, and 0.6% (95% CI: 0.3%- 1.0%) were comorbid with three other mental disorders. The co-occurrence of anxiety disorder and ADHD, conduct disorder (CD) and oppositional defiant disorder (ODD) was very common. The most common comorbidity of GAD, specific phobia, and separation anxiety disorder was ODD, with comorbid rates of 26.9%, 74.7%, and 49%, respectively. The most common comorbidity for panic

Confidential: For Review Only

disorder was CD (24%). The ADHD-inattention subtype ranks as the most prevalent comorbidity for agoraphobia. Tic disorder was also common in anxiety disorder, and the comorbid rate of provisional tic disorder was 5.2% for GAD, 7.2% for panic disorder, and 10.8% for specific phobia. Tourette's syndrome and chronic vocal tic disorder were also common in agoraphobia. The concurrent rates were 10.1% and 6.3%, respectively. In our survey, there was no comorbidities of anxiety disorder and eating disorder, adjustment disorder, psychotic disorders, or pervasive developmental disorder (for details, please refer to figure 3).

Insert Figure 3

4. Discussion

This first national survey carried out in China showed that GAD was the most common anxiety disorder in school children and adolescents, with a prevalence rate of 1.3%, followed by social phobia (0.9%), separation anxiety disorder (0.6%), panic disorder (0.2%), specific phobia (0.2%), and agoraphobia (0.1%). The distribution of anxiety disorder was different among different age and gender group. Children had higher prevalence rate of separation anxiety disorder and specific phobia than adolescents. Girls were more likely to be affected by panic disorder and GAD than boys. As for comorbidities, attention-deficit and disruptive behavior disorders, tic disorders and depressive disorders were also commonly observed in the pediatric anxiety population.

Previous studies have shown that the prevalence of anxiety disorders in the pediatric population varies from 2% to 31% in different countries[8–11,16,17]. The prevalence of any anxiety disorder in our study was 4.7%. Our result was compatible with two other surveys carried out in Hunan and Sichuan provinces in China[9,16]. The prevalence rate was lower than that in many other studies[11,17,18]. The main reason might be related to the diagnostic criteria. In our survey, the DSM-IV criteria were used for the final diagnosis, which required functional impairment when making the diagnosis. A previous study showed that the prevalence of psychiatric disorders decreased sharply when the impairment criteria were applied[18]. In addition, stigma

Confidential: For Review Only

might be associated with low prevalence, as people in Asian countries are not used to reporting emotional problems[27]. Children and adolescents with self-stigma might not report their anxious experience[28]. As anxiety is an internal symptom, it might be ignored by caregivers.

Among the subtypes of anxiety disorder, GAD was the most prevalent anxiety disorder in our study (1.3%), which was consistent with two studies carried out in Hunan and Sichuan provinces, China. By investigating 17,071 primary and middle school students in Central Hunan Province, Shen et al reported that the 12-month prevalence of GAD was 1.77%, followed by 0.74% for a specific phobia, 0.07% for social phobia, and 0.02% for social anxiety disorder[9]. Roberts et al reported that agoraphobia was the most common anxiety disorder in their sample[29]. Several other studies reported that specific anxiety disorders had a much higher prevalence rate than other anxiety disorders[11,17]. However, the prevalence of panic disorder was relatively low (0.2%) in our study and other studies[11,16]. Notably, panic attacks are very common in the pediatric population. In the survey by Essau et al, 18% of adolescents reported experiencing panic attacks. However, only 0.5% met the diagnosis of panic disorder[3].

Both age and gender had effects on the prevalence of any anxiety disorder. Our results showed that girls were more likely to be affected by GAD and panic disorder. Gender differences have been reported in many previous studies[8,9,16]. Wagner et al reported that the prevalence rate of anxiety disorder was one time higher in girls than in boys (19.53 and 9.52%, respectively)[10]. However, some other studies found no gender difference[1,2]. The distribution of anxiety disorders was also different in different age groups. In general, adolescents had a higher rate of anxiety disorder, except for separation anxiety disorder. This was consistent with previous findings that anxiety disorder was less prevalent in younger children[11,30]. In our study, the peak age for separation anxiety disorder was between 7 and 10 years old. The finding was similar to that of Mohammadi et al[1]. This might be related to the fact that at this time, children separated from their parents and started attending school. Most previous studies have shown that young children have a higher prevalence rate of separation

Confidential: For Review Only

anxiety disorder[1,2]. In contrast, the prevalence of social anxiety disorder increases with age[30]. Our results found no difference between developed areas and less developed areas. The effect of socioeconomic factors on the prevalence of anxiety disorder is not clear. A cross-nation survey indicated that socioeconomic conditions might be associated with the differences in the prevalence of anxiety subtypes, and high-income countries had higher prevalence rates of social anxiety disorder than low- and lower-middle income countries[31]. Many other studies failed to find differences between different socioeconomic status groups[10,16].

In our study, 79.8% of children with anxiety disorder had at least one comorbidity. Previous studies indicated that the comorbid rate of anxiety disorder was high[32]. The comorbidity of other subtypes of anxiety disorder, depressive disorder, ADHD and disruptive, impulse control and conduct disorders was common[2,10,30]. Our results showed that the most prevalent comorbidities of anxiety disorder were disruptive, impulse control and conduct disorders. The most common comorbidity for GAD, specific phobia and separation anxiety disorder was ODD. CD ranked as the most common comorbidity for panic disorder. The high rate of comorbidity might be due to the following reasons. First, the symptoms of anxiety disorder and ODD and CD were highly associated[33]. Anger or irritability symptoms in the ODD lead to anxiety[34]. In addition, the functional impairment caused by ODD increased the risk of anxiety[35]. The concurrence of anxiety disorder and disruptive, impulse control and conduct disorders would cause more severe functional impairment for the individual [36]. Comorbidity with ADHD was also very common in our study. The comorbidity of anxiety disorder and ADHD could be explained by the attentional control theory[37]. Individuals in an anxious state have increased attention to threat-related stimuli and impaired attentional inhibition and shifting skills.[38]. Executive attention was impaired in children with anxiety disorders[39]. A neuroimaging study also confirmed deficits in prefrontal attentional control mechanisms in anxiety patients[40].

The study had several limitations. First, the participants were enrolled in schools. Children who did not attend school were not included, and some of them might may not attend school because of anxiety. This would undermine the representativeness of

Confidential: For Review Only

our sample. Second, the MINI-KID and the DSM-IV criteria were used for the diagnosis. However, the validity of the MINI-KID for the diagnosis of affective disorders, such as dysthymia, was not as good[25]. This may make the comorbidity results less comprehensive. The DSM-IV rather than DSM-5 diagnostic criteria was used in this study as it was started in October 2014, and the Chinese version of DSM-5 was published in July 2014. There was not enough time and resource to train clinicians to perform the DSM-5 assessment. The classification of anxiety disorder subtypes had changed a lot in DSM-5 criteria. For example, selective mutism was categorized as anxiety disorder in DSM-5 but not in DSM-IV. This would limit the comparability of the results with recent studies based on DSM-5. Thirdly, it should be noted that all diagnoses were made retrospectively, and recall bias could be a confounding factor for the results. Although according to the DSM-IV diagnostic criteria, functional impairment was considered while made the final diagnosis, there still lacks explicit measurement for functional impairment using psychological scales such as clinical global impression (CGI), this is another limitation of the study.

This study was a two-stage, national epidemiological survey using a stratified cluster sampling method. The participants represented school children and adolescents from different socio-economic backgrounds. The results demonstrated that anxiety disorder was common among school students. Girls were more likely to be affected by anxiety disorder than boys. The distribution of anxiety changed with age. Caregivers and teachers should pay more attention to the risk emotions of children. Our results also revealed that ADHD, ODD and CD often co-occurred with anxiety disorder. This implies that for children existing external symptoms such as aggressive symptoms and hyperactivity, the assessment of internal symptoms should not be neglected. A comprehensive assessment should be carried out in case the anxiety disorder is masked by external symptoms.

Funding statement: This work was supported by the National Natural Science Foundation of China (NSFC) under Grant No. 82001445, No. 82171538, the Natural Science Foundation of Beijing Municipality under Grant No. 7212035, Beijing

Confidential: For Review Only

Hospitals Authority Youth Programme Grant No. QML20211203 and Beijing Chronic Disease Prevention and Health Education Research Programme under Grant No. BJMB0012022028019.

Competing interests: The authors declare no competing interests.

Patient and public involvement: Patients and/or the public were not involved in the design, or conduct, or reporting, or dissemination plans of this research.

Roles of authors: Yi Zheng and Yonghua Cui was responsible for the design and carry-out of the survey. Fang Wang contributed to the data analysis and writing of the manuscript. Hui Xu and Ying Li collaborated in the writing of the manuscript. Rui Wang, Fenghua Li and Ying Li participated in collecting data collection and digitalizing the data.

Confidential: For Review Only

Reference：

1. Mohammadi MR, Ahmadi N, Yazdi FR, et al. Prevalence, comorbidity and predictors of anxiety disorders among children and adolescents. Asian J Psychiatr 2020;53:102059.doi: 10.1016/j.ajp.2020.102059.

2. Spence SH, Zubrick SR, Lawrence D. A profile of social, separation and generalized anxiety disorders in an Australian nationally representative sample of children and adolescents: Prevalence, comorbidity and correlates. Aust N Z J Psychiatry 2018;52:446–460. doi: 10.1177/0004867417741981.

3. Essau CA, Conradt J, Petermann F. Frequency, comorbidity, and psychosocial impairment of anxiety disorders in German adolescents. J Anxiety Disord 2000;14:263–279 .doi:10.1016/s0887-6185(99)00039-0.

4. Hossain B, Bent S, Hendren R. The association between anxiety and academic performance in children with reading disorder: A longitudinal cohort study. Dyslexia 2021;27:342–354.doi: 10.1002/dys.1680.

5. Shek DTL, Chai W, Tan L. The relationship between anxiety and depression under the pandemic: The role of life meaning. Front Psychol 2022;13:1059330.doi: 10.3389/fpsyg.2022.1059330.

6. Woodward LJ, Fergusson DM. Life course outcomes of young people with anxiety disorders in adolescence. J Am Acad Child Adolesc Psychiatry 2001;40:1086–1093.doi: 10.1097/00004583-200109000-00018.

7. Richardson LP, Russo JE, Lozano P, et al. Factors associated with detection and receipt of treatment for youth with depression and anxiety disorders. Acad Pediatr 2010;10:36–40. doi: 10.1016/j.acap.2009.09.011.

8. Ravens-Sieberer U, Wille N, Erhart M, et al. Prevalence of mental health problems among children and adolescents in Germany: results of the BELLA study within the National Health Interview and Examination Survey. Eur Child Adolesc Psychiatry 2008;17 Suppl 1:22–33. doi: 10.1007/s00787-008-1003-2.

9. Shen Y-M, Chan BSM, Liu J-B, et al. The prevalence of psychiatric disorders among students aged 6~16 years old in central Hunan, China. BMC Psychiatry 2018;18:243. doi: 10.1186/s12888-018-1823-7.

10. Wagner G, Zeiler M, Waldherr K, et al. Mental health problems in Austrian adolescents: a nationwide, two-stage epidemiological study applying DSM-5 criteria. European Child & Adolescent Psychiatry 2017;26:1483. doi: 10.1007/s00787-017-0999-6.

11. Merikangas KR, He J-P, Burstein M, et al. Lifetime prevalence of mental disorders in U.S. adolescents: results from the National Comorbidity Survey Replication--Adolescent

Confidential: For Review Only

Supplement (NCS-A). J Am Acad Child Adolesc Psychiatry 2010;49:980–989. doi: 10.1016/j.jaac.2010.05.017.

12. Walkup JT, Albano AM, Piacentini J, et al. Cognitive behavioral therapy, sertraline, or a combination in childhood anxiety. N Engl J Med 2008;359:2753–2766. doi: 10.1056/NEJMoa0804633.

13. Choi EPH, Hui BPH, Wan EYF. Depression and Anxiety in Hong Kong during COVID-19. Int J Environ Res Public Health 2020;17:3740. doi: 10.3390/ijerph17103740.

14. D'Agati E, Curatolo P, Mazzone L. Comorbidity between ADHD and anxiety disorders across the lifespan. Int J Psychiatry Clin Pract 2019;23:238–244. doi: 10.1080/13651501.2019.1628277.

15. Ghandour RM, Sherman LJ, Vladutiu CJ, et al. Prevalence and Treatment of Depression, Anxiety, and Conduct Problems in US Children. J Pediatr 2019;206:256-267.e3. doi: 10.1016/j.jpeds.2018.09.021.

16. Qu Y, Jiang H, Zhang N, et al. Prevalence of Mental Disorders in 6–16-Year-Old Students in Sichuan Province, China. Int J Environ Res Public Health 2015;12:5090–5107. doi: 10.3390/ijerph120505090.

17. Chen Y-L, Chen WJ, Lin K-C, et al. Prevalence of DSM-5 mental disorders in a nationally representative sample of children in Taiwan: methodology and main findings. Epidemiol Psychiatr Sci 2019;29:e15. doi:10.1017/S2045796018000793.

18. Leung PWL, Hung S, Ho T, et al. Prevalence of DSM-IV disorders in Chinese adolescents and the effects of an impairment criterion: a pilot community study in Hong Kong. Eur Child Adolesc Psychiatry 2008;17:452–461. doi: 10.1007/s00787-008-0687-7.

19. Li F, Cui Y, Li Y, et al. Prevalence of mental disorders in school children and adolescents in China: diagnostic data from detailed clinical assessments of 17,524 individuals. 2021;13:34-46. doi: 10.1111/jcpp.13445.

20. Huang Y, Wang Y, Wang H, *et al.* Prevalence of mental disorders in China: a cross-sectional epidemiological study. *Lancet Psychiatry*. 2019;6:211–24.

21. Centers for Disease Control and Prevention (CDC). Prevalence of diagnosed Tourette syndrome in persons aged 6-17 years - United States, 2007. MM1 Mohammadi MR, Ahmadi N, Yazdi FR, *et al.* Prevalence, comorbidity and predictors of anxiety disorders among children and adolescents. *Asian J Psychiatr*. 2020;53:102059.

22. Leung PWL, Kwong SL, Tang CP, et al. Test-retest reliability and criterion validity of the Chinese version of CBCL, TRF, and YSR. J Child Psychol Psychiatry 2006;47:970–973. doi: 10.1111/j.1469-7610.2005.01570.x.

Confidential: For Review Only

23. Seligman LD, Ollendick TH, Langley AK, et al. The utility of measures of child and adolescent anxiety: a meta-analytic review of the Revised Children's Manifest Anxiety Scale, the State-Trait Anxiety Inventory for Children, and the Child Behavior Checklist. J Clin Child Adolesc Psychol 2004;33:557–565. doi: 10.1207/s15374424jccp3303_13.

24. Liu X, Kurita H, Guo C, et al. Prevalence and risk factors of behavioral and emotional problems among Chinese children aged 6 through 11 years. J Am Acad Child Adolesc Psychiatry 1999;38:708–715. doi: 10.1097/00004583-199906000-00018.

25. Sheehan DV, Sheehan KH, Shytle RD, et al. Reliability and validity of the Mini International Neuropsychiatric Interview for Children and Adolescents (MINI-KID). J Clin Psychiatry 2010;71:313–326. doi: 10.4088/JCP.09m05305whi.

26. Rao, J., & Scott, A. On simple adjustments to chi-square tests with sample survey data. The Annals of Statistics 1987;15:385-397. doi: 10.1214/aos/1176350273.

27. Lynch H, McDonagh C, Hennessy E. Social Anxiety and Depression Stigma Among Adolescents. J Affect Disord 2021;281:744–750. doi: 10.1016/j.jad.2020.11.073.

28. Calear AL, Batterham PJ, Torok M, et al. Help-seeking attitudes and intentions for generalised anxiety disorder in adolescents: the role of anxiety literacy and stigma. Eur Child Adolesc Psychiatry 2021;30:243–251. doi: 10.1007/s00787-020-01512-9.

29. Roberts RE, Roberts CR, Xing Y. Rates of DSM-IV Psychiatric Disorders Among Adolescents in a Large Metropolitan Area. J Psychiatr Res 2007;41:959–967. doi: 10.1016/j.jpsychires.2006.09.006.

30. Cummings CM, Caporino NE, Kendall PC. Comorbidity of anxiety and depression in children and adolescents: 20 years after. Psychol Bull 2014;140:816–845. doi: 10.1037/a0034733.

31. Stein DJ, Lim CCW, Roest AM, et al. The cross-national epidemiology of social anxiety disorder: Data from the World Mental Health Survey Initiative. BMC Med 2017;15:143. doi: 10.1186/s12916-017-0889-2.

32. Rapee RM, Lyneham HJ, Hudson JL, et al. Effect of comorbidity on treatment of anxious children and adolescents: results from a large, combined sample. J Am Acad Child Adolesc Psychiatry 2013;52:47–56. doi: 10.1016/j.jaac.2012.10.002.

33. Humphreys KL, Aguirre VP, Lee SS. Association of anxiety and ODD/CD in children with and without ADHD. J Clin Child Adolesc Psychol 2012;41:370–377. doi: 10.1080/15374416.2012.656557.

34. Drabick DAG, Gadow KD. Deconstructing oppositional defiant disorder: clinic-based evidence for an anger/irritability phenotype. J Am Acad Child Adolesc Psychiatry 2012;51:384–393. doi: 10.1016/j.jaac.2012.01.010.

Confidential: For Review Only

35. Evans SC, Burke JD, Roberts MC, et al. Irritability in child and adolescent psychopathology: An integrative review for ICD-11. Clin Psychol Rev 2017;53:29–45. doi: 10.1016/j.cpr.2017.01.004.

36. Martín V, Granero R, Ezpeleta L. Comorbidity of oppositional defiant disorder and anxiety disorders in preschoolers. Psicothema 2014;26:27–32. doi: 10.7334/psicothema2013.75.

37. Eysenck MW, Derakshan N, Santos R, et al. Anxiety and cognitive performance: attentional control theory. Emotion 2007;7:336–353. doi: 10.1037/1528-3542.7.2.336.

38. Ironside M, Browning M, Ansari TL, et al. Effect of Prefrontal Cortex Stimulation on Regulation of Amygdala Response to Threat in Individuals With Trait Anxiety. JAMA Psychiatry 2019;76:71–78. doi: 10.1001/jamapsychiatry.2018.2172.

39. Mogg K, Salum GA, Bradley BP, et al. Attention network functioning in children with anxiety disorders, attention-deficit/hyperactivity disorder and non-clinical anxiety. Psychol Med 2015;45:2633–2646. doi: 10.1017/S0033291715000586.

40. Forster S, Nunez Elizalde AO, Castle E, et al. Unraveling the Anxious Mind: Anxiety, Worry, and Frontal Engagement in Sustained Attention Versus Off-Task Processing. Cereb Cortex 2015;25:609–618. doi: 10.1093/cercor/bht248.

Confidential: For Review Only

Figure legend

Figure 1. The flowchart of the study.

Figure 2. The prevalence rate of anxiety disorder at different age.

Figure 3. Comorbid conditions of anxiety disorder.

Table 1. The point prevalence of different subtypes of anxiety disorder.

Table 2. The prevalence rate of anxiety disorder in children and adolescents group.

Table 3. The prevalence rate of anxiety disorder in different gender group.

Table 4. The prevalence rate of anxiety disorder in developed and underdeveloped areas.

Supplementary file 1. The prevalence rate of anxiety disorder at different age.

Confidential: For Review Only

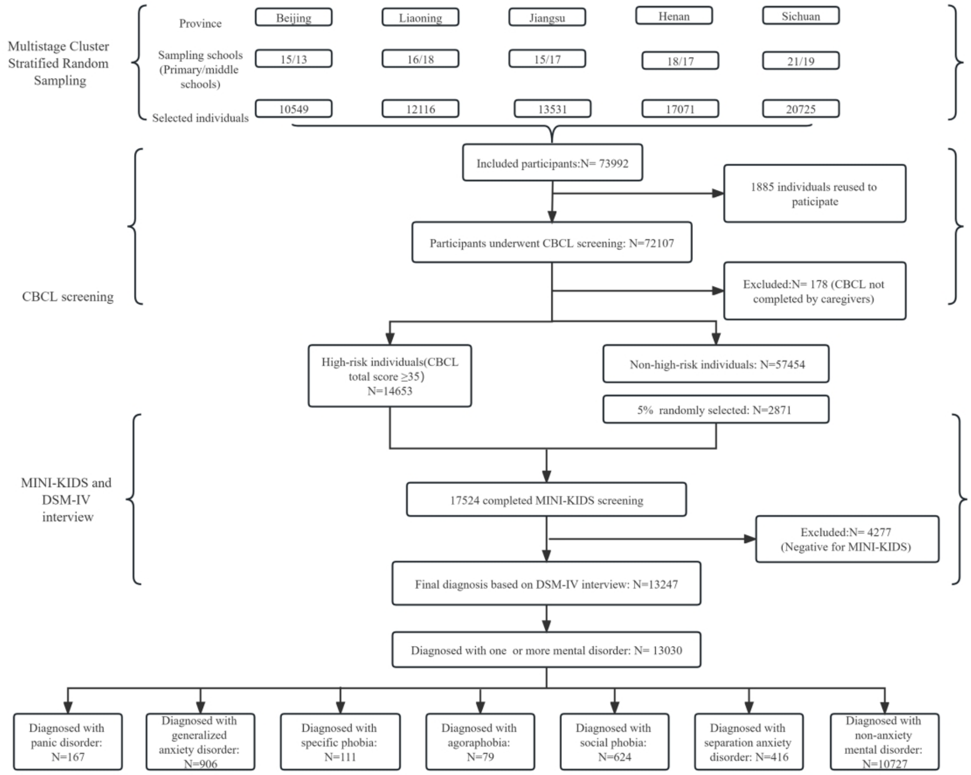

The flowchart to identify included participants

209x169mm (300 x 300 DPI)

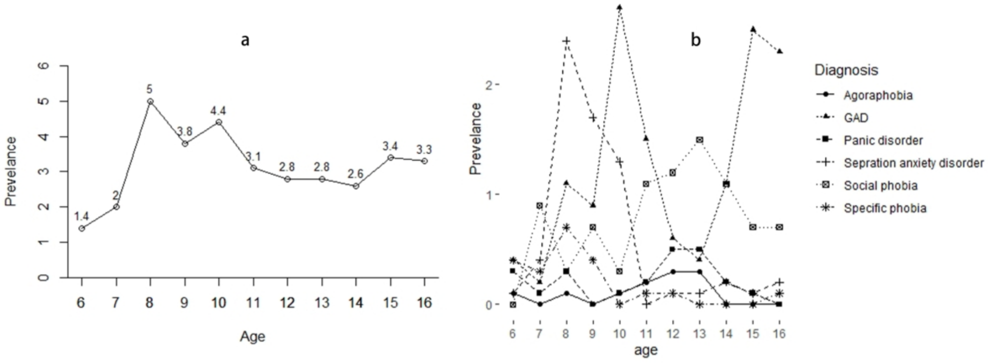

The prevalence of anxiety disorders change with the age

209x77mm (300 x 300 DPI)

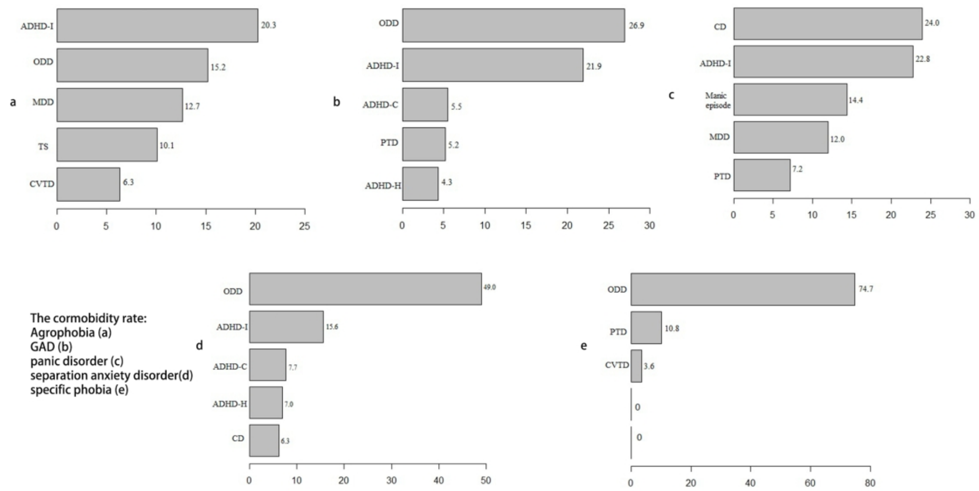

The comorbidity rate of anxiety disorders

209x107mm (300 x 300 DPI)

Supplementary File 1. The prevalence rate of anxiety disorder at different age.

Anxiety disorder \ Age	6	7	8	9	10	11	12	13	14	15	16
Panic disorder	0.3	0.1	0.3	0	0.1	0.2	0.5	0.5	0.2	0.1	0
Agoraphobia without panic	0.1	0	0.1	0	0.1	0.2	0.3	0.3	0	0	0
Separation anxiety disorder	0.1	0.4	2.4	1.7	1.3	0	0.1	0.1	0.2	0.1	0.1
Social phobia	0	0.9	0.3	0.7	0.3	1.1	1.2	1.5	1.1	0.7	0.5
Specific phobia	0.4	0.3	0.7	0.4	0	0.1	0.1	0	0	0	0.1
Generalized Anxiety Disorder	0.4	0.2	1.1	0.9	2.7	1.5	0.6	0.4	1.1	2.5	2.2
Total	1.4	2.0	5.0	3.8	4.4	3.1	2.8	2.8	2.6	3.4	3.0

Confidential: For Review Only
